# An antisense promoter in mouse L1 retrotransposon open reading frame-1 initiates expression of diverse fusion transcripts and limits retrotransposition

**DOI:** 10.1093/nar/gku091

**Published:** 2014-01-31

**Authors:** Jingfeng Li, Manoj Kannan, Anna L. Trivett, Hongling Liao, Xiaolin Wu, Keiko Akagi, David E. Symer

**Affiliations:** ^1^Department of Molecular Virology, Immunology and Medical Genetics, The Ohio State University, Columbus, OH 43210, USA, ^2^Basic Research Laboratory, Center for Cancer Research, National Cancer Institute, Frederick, MD 21702, USA, ^3^Laboratory of Molecular Technology, Advanced Technology Program, SAIC-Frederick, Inc., Frederick, MD 21702, USA, ^4^Mouse Cancer Genetics Program, Center for Cancer Research, National Cancer Institute, Frederick, MD 21702, USA, ^5^Human Cancer Genetics Program, The Ohio State University Comprehensive Cancer Center, Columbus, OH 43210, USA, ^6^Internal Medicine, The Ohio State University, Columbus, OH 43210, USA and ^7^Biomedical Informatics, The Ohio State University, Columbus, OH 43210, USA

## Abstract

Between 6 and 30% of human and mouse transcripts are initiated from transposable elements. However, the promoters driving such transcriptional activity are mostly unknown. We experimentally characterized an antisense (AS) promoter in mouse L1 retrotransposons for the first time, oriented antiparallel to the coding strand of L1 open reading frame-1. We found that AS transcription is mediated by RNA polymerase II. Rapid amplification of cDNA ends cloning mapped transcription start sites adjacent to the AS promoter. We identified >100 novel fusion transcripts, of which many were conserved across divergent mouse lineages, suggesting conservation of potential functions. To evaluate whether AS L1 transcription could regulate L1 retrotransposition, we replaced portions of native open reading frame-1 in donor elements by synonymously recoded sequences. The resulting L1 elements lacked AS promoter activity and retrotransposed more frequently than endogenous L1s. Overexpression of AS L1 transcripts also reduced L1 retrotransposition. This suppression of retrotransposition was largely independent of Dicer. Our experiments shed new light on how AS fusion transcripts are initiated from endogenous L1 elements across the mouse genome. Such AS transcription can contribute substantially both to natural transcriptional variation and to endogenous regulation of L1 retrotransposition.

## INTRODUCTION

Long interspersed elements (LINEs, L1s) are a major class of mammalian retrotransposons, comprising ∼19 and 21% of the mouse and human genomes, respectively (1,2). Approximately half of the mammalian genome has resulted from L1-mediated mobilization (3). Ongoing, endogenous L1 retrotransposition has caused widespread genomic structural variation between mouse strains (4,5) and between human individuals (6,7), and also causes somatic variation both in normal development and in certain human cancers (8). Full-length L1s (∼6.0 kilobases, in human, ∼7.0 kb in mouse) contain an internal sense-stranded promoter in the 5′ untranslated region (UTR), two open reading frames (ORF1 and ORF2) and a 3′ UTR with a poly(A) tail (3). ORF1 encodes a nucleic acid-binding chaperone protein (9,10), whereas ORF2 encodes an endonuclease (11), reverse transcriptase and a zinc finger-like protein (12). Both ORFs are required for autonomous retrotransposition (13). Thousands of full-length elements in three young L1 subfamilies (T_F_, G_F_ and A) reside in the mouse genome (4,14,15). The mouse L1 subfamilies are defined by differences in their 5′ UTR monomeric repeats. ORF2 contains the fewest nucleotide variants, whereas the 3′ UTR has the most (15). Members of each subfamily have integrated into the mouse genome after the evolutionary split between rat and mouse. Many L1 T_F_, G_F_ and A integrants are polymorphic, reflecting recent ongoing retrotransposition (4,5,15).

The myriad potential biological impacts of endogenous transposable elements (TEs) in human and mouse appear to depend on their genomic context, their sequence structure and other factors (16). Endogenous TEs have been shown to affect neighboring gene expression in various ways. For example, they have been reported to initiate a surprising number, between 6 and 30%, of human and mouse transcripts (17). In humans, an active antisense (AS) L1 promoter in the 5′ UTR of full-length L1s initiates expression of numerous distinct AS L1 retrotransposon-initiated fusion transcripts (RIFTs), thereby contributing to and modifying the expression of numerous neighboring genes (18–22). As a majority of full-length intragenic human L1s are oriented AS to flanking genes’ ORFs (23,24), resulting AS L1 RIFTs frequently include downstream spliced exons expressed in the canonical sense orientation. Other human AS L1 RIFTs are noncoding (25–27). Mouse endogenous retroviruses have been shown to disrupt overlapping gene expression (28,29). Human L1s may affect expression of overlapping genes, including the *Met* proto-oncogene (30) and others (31).

Like other mammalian TEs, L1s are constrained by various cellular defenses including DNA methylation, histone modifications, Dicer-mediated RNA interference (RNAi) and other small RNA-mediated effects (32–37). Bidirectional promoters within the human L1 5′ UTR, i.e. the internal sense and AS promoters (20), ∼500 nucleotides apart, can initiate double-stranded transcripts that can be processed to small interfering RNAs (siRNAs) by Dicer (34,38). Single-stranded transcripts also can be processed to small RNAs, regardless of whether they are initiated within or outside of L1 elements. Resulting L1-specific small RNAs could mediate transcriptional and/or posttranscriptional gene silencing (34,36,39–42). Both sense and AS transcripts mapping to the 5′ end of full-length mouse L1 elements are expressed in mouse embryonic stem (ES) cells (43,44). Mouse chimeric transcripts containing AS L1 sequences also have been identified (4,45). Together, these results suggest that mouse L1 elements also may contain one or more AS promoters. However, despite identification of AS L1 RIFTs in mouse testis and of sense and AS transcripts in mouse ES cells, a putative AS promoter has not been experimentally validated up to now. Moreover, both its activity in other tissues and its possible biological roles have not been described. Here, we identified an active mouse AS L1 promoter within ORF1, immediately proximal to AS L1 RIFTs’ transcription start sites (TSS). We found that the resulting AS mouse L1 RIFTs, including spliced, unspliced and many noncoding RNAs, were initiated by interspersed L1s genome-wide. Our results indicate that AS L1 RIFTs contribute to the diverse transcriptome (including long noncoding RNAs) expressed in various tissues (25,26,46). AS transcription also helps to limit mouse L1 retrotransposition through a Dicer-independent mechanism (42).

## MATERIALS AND METHODS

### Mouse colony, cell lines and isolation of genomic DNA and RNA

Mice were maintained and euthanized according to approved Institutional Animal Care and Use Committee protocols (National Cancer Institute, Frederick, MD, USA; Ohio State University, Columbus, OH, USA). Mouse strains and purified genomic DNA were purchased from the Jackson Laboratory (Bar Harbor, ME, USA). A mouse spermatocyte cell line (CRL2196) was purchased from the American Type Culture Collection. HeLa cells were provided by Dr John V. Moran (University of Michigan). HCT116 *Dicer* ex5 knockout cells were provided by Dr Bert Vogelstein (Johns Hopkins).

Genomic DNA and pooled total RNAs were isolated from CRL2196 cells and from various tissues, ages and lineages of mice as indicated, using standard methods and Trizol (Invitrogen), respectively.

### Oligonucleotide sequences

Primer sequences and annotations are listed in Supplementary Table S1.

### Candidate promoter activity assays

**Figure 1.**
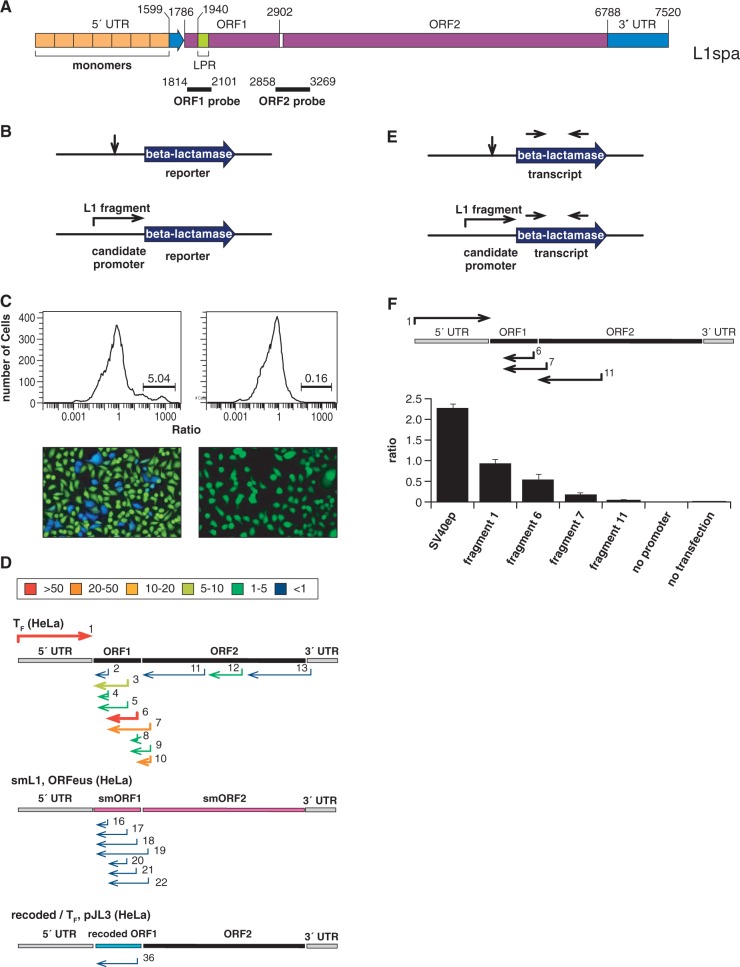
Mapping an active AS promoter within L1 ORF1. (**A**) Schematic representation of an L1 T_F_ subfamily retrotransposon, L1spa, with coordinates indicated as used throughout this article. L1spa was identified in GenBank accession no. AF016099. Below: Probes for phage cDNA library hybridization against ORF2 (2858–3269 nt) and ORF1 (1814–2101 nt). (**B**) Various DNA fragments were directionally engineered upstream of a promoterless reporter gene, i.e. β-lactamase *TEM1*. (**C**) Linearized DNAs containing various candidate promoter-reporter cassettes were transfected into HeLa cells. Functional beta-lactamase protein expression was measured by staining cells with CCF2-AM, whose fluorescence emission shifts from green to blue on increased enzymatic cleavage (48). Cells expressing (left) or not expressing (right) β-lactamase were evaluated both by flow cytometry (top), which measured quantitative blue/green emission ratios (49), and by fluorescence microscopy (bottom). (**D**) Fragments derived from various L1 positions and subclasses were numbered and directionally oriented as indicated (Supplementary Figure S1 and Supplementary Table S1). Their promoter strengths were assayed as described in part B. Key: colors and thicknesses indicate promoter activity scores for each fragment assayed. The highest scores (>50, red, thick line) indicate strongest promoter activities. (**E**) TEM1 transcript levels were measured using qRT-PCR (arrows: primer binding sites) to assess the candidate fragments’ promoter activities. (**F**) The ratio of *TEM1* to beta-actin transcript concentrations was calculated (*y-axis*) after correction for amplification of contaminating plasmid or genomic DNA. As a positive control, SV40 early promoter was engineered upstream of the *TEM1* reporter, and as negative controls, no promoter was included or no plasmid was transfected. The AS L1 promoter activity (fragment 6) is half that of the sense-stranded mouse L1 5′ UTR promoter (fragment 1). Fragments are numbered as in (D).

Genomic DNA fragments representing four mouse L1 subfamilies (14) (T_F_ GenBank accession number AF016099; G_F_, AC068252; A, AY053456 and FIII, AC002406) and a synthetic L1 element smL1 (47) were amplified by PCR using Platinum Taq HiFi (Invitrogen) and forward and reverse primers incorporating *BglII* and *NcoI* restriction sites, respectively (Supplementary Table S1). Amplicons included fragments of L1 T_F_ (represented by L1spa in pTN201), sense promoter (primers DES1212 and DES1213), AS promoter (DES1218 × DES1220, DES1218 × DES1221) and AS ORF2 (DES1459 × DES1460). Promoter candidates were cloned directionally upstream of *TEM1*, a β-lactamase reporter gene, in pBLAK-b, which lacks a promoter (Invitrogen) (Figure 1 and Supplementary Figure S1). They were confirmed by sequencing. One microgram of *Bgl*II-digested (linearized) plasmid DNA was transiently transfected into CRL2196 or HeLa cells using FuGENE 6 (Roche). As positive and negative controls, plasmids with and without the SV40 promoter upstream of *TEM1* were used (pBLAK-c and pBLAK-b, respectively).

To quantify β-lactamase protein expression, cells were stained with CCF2/AM substrate (Invitrogen) (48,49) by replacing culture medium with 1 ml loading solution [2 µl of a 1-mM CCF2/AM solution, 16 µl of solution B, 10 µl of 250 mM Probenicid (Sigma) and 972 µl Hanks’ Balanced Salt solution, (HBSS)] per 9.6 cm^2^ well. Cells were incubated in the dark at room temperature (RT) for 1 h with gentle shaking, washed with HBSS and visualized using an Axiovert 200 M inverted fluorescence microscope (Zeiss) with blue/aqua and β-lactamase ratio filter sets (Chroma Technology Corp.) and ORCA-ER high resolution digital camera (Hamamatsu Photonics) using Openlab software (version 4.0.2, Improvision). Flow cytometric analysis was performed using a BD LSR II flow cytometer with a 405 nm violet laser, 440/40 nm (blue) and 530/30 nm (green) filters, and FACSDiva software (BD Biosciences). Ratios of blue to green intensities were collected as a linear parameter. Each flow cytometry session included positive and negative controls to normalize output.

*TEM1* expression also was quantified by quantitative reverse transcriptase-mediated PCR (qRT-PCR). Promoter candidates were linearized by *Bgl*II digestion and transfected into HeLa cells using FuGENE 6. Total RNAs were isolated ∼48 h after transfection using RNeasy kit (Qiagen). Standard curves were based on serial dilutions of control plasmids. First strand cDNAs were synthesized using oligo-d(T) (DES2633) primer and the SuperScript double-stranded cDNA synthesis kit (Invitrogen). As further controls, RNAs were treated with and without reverse transcriptase. qRT-PCR was performed on an iCycler (Bio-Rad) or Step One Plus (Applied Biosystems) instrument, using SYBR Green Supermix (Bio-Rad). *TEM1* transcript concentrations were calculated by interpolation, after subtracting for input plasmid DNA contamination. Beta-actin transcript levels were calculated for each sample. Each sample was measured in triplicate. Results are presented for each sample as the normalized ratio of *TEM1* to beta-actin transcript levels.

### Chromatin immunoprecipitation of RNA polymerases

Anti-mouse RNA polymerase III subunit RPC39 mouse monoclonal antibody was purchased from Santa Cruz (catalog no. SC-21753). Anti-mouse RNA polymerase II mouse monoclonal antibody (Cat. 39097) was from Active Motif. For chromatin immunoprecipitation (ChIP), the Magna ChIP G Tissue kit (Millipore) was used following the manufacturer’s instructions.

### Identification of TSS of TE-initiated fusion transcripts

We performed 5′ RACE cloning using a second-generation 5′/3′ RACE kit (Roche) with an AS L1 ORF1-specific primer (DES1947; cf. Supplementary Table S1) for first strand cDNA synthesis.

### Phage library screens for mouse transcripts containing L1 sequences

Double-stranded DNA probes for mouse L1 ORF2 and ORF1 transcripts were amplified by PCR from L1spa (AF016099), a representative full-length T_F_ template (50). Bacteriophage cDNA libraries from mouse testis (Clontech) and thymus (Stratagene) were hybridized with an ORF2 probe followed by an ORF1 probe.

The primer pairs used to amplify fragments from L1spa ORF2 and ORF1 were DES1165 × DES1166 and DES 1167 × DES1168, respectively (Supplementary Table S1). Resulting PCR products were gel purified and radiolabeled by random nonamer priming. Commercial bacteriophage cDNA libraries from mouse testis (Clontech) and thymus (Stratagene) were plated at ∼50 000 plaques per dish, transferred to Hybond-N filters (Amersham) and hybridized with the ORF2 probe. Filters were washed at 65°C in 0.1 × SSC and 0.1% SDS and autoradiographed. This procedure was repeated with the ORF1 probe to identify ORF1^+^ORF2^−^ clones, which were purified upon additional rounds of hybridization. Phage plaques were converted to plasmids and sequenced using BigDye v. 3.1 (Applied Biosystems) on a 96 capillary sequencer (Transgenomic Spectrumedix) with primers DES886 and DES837 (5′ and 3′ ends, testis cDNA) and standard M13R and M13F oligonucleotides (5′ and 3′ ends, thymus). Additional sequences for full-length cDNA sequencing by primer walking are available on request.

### RT-PCR amplification of AS L1 RIFTs

To target AS L1 RIFTs, first strand cDNAs were synthesized (Roche) from DNase-treated total RNAs, using the M13F-anchored primer DES1141 paired with DES1256 for mouse L1spa ORF1, AS nucleotides 2011–1991. PCR products (1–3 kb) were isolated using gel purification columns (Qiagen), and cloned for sequencing. Tissue-specific expression patterns of AS L1 RIFTs and corresponding cognate genes were analyzed by RT-PCR using a commercial multiple-tissue mouse cDNA panel (Clontech).

### Computational identification of AS L1 RIFTs

A BLASTN search of mouse EST databases from testis and other tissues was conducted using AS L1spa (T_F_ subfamily) ORF1 as query, i.e. AS nucleotides 2225–1838 (cf. coordinates, Figure 1).

### Identification of RIFTs using exon microarrays

To develop a novel assay to identify L1 RIFTs, we modified the manufacturer’s protocol for the Affymetrix GeneChip mouse exon microarray. First strand cDNA synthesis was performed on total RNA isolated from various tissues and lineages, using a primer including both T7 promoter and oligo-d(T) sequences and SuperScript II reverse transcriptase (Invitrogen). Polyadenylated cDNAs containing AS L1 sequences were amplified using a primer for a particular L1 ORF1 AS template sequence paired with the T7 promoter primer. Resulting double-stranded RIFT cDNAs containing T7 sequences were used as templates for *in vitro* transcription, following standard procedures (Affymetrix). Resulting AS RNA was purified; a second round of first strand cDNA synthesis was performed with reverse transcriptase, dUTP and random primers; cRNA was hydrolyzed using RNaseH, and resulting sense strand DNA was purified. Products were fragmented with uracil DNA glycosylase and apurinic/apyrimidinic (AP)-endonuclease I. Terminal labeling was performed with terminal deoxynucleotidyl transferase (TdT), and resulting labeled fragments were hybridized to the exon microarray.

Resulting raw data from an Affymetrix microarray chip reader were analyzed for transcript expression, using Partek Genomics Suite software. CEL files (MoEx-1_0-st-v1) were imported using RMA background correction and quantile normalization. Probe intensities were transformed to log base 2. We defined signals with intensity > mean + one standard deviation (i.e. ∼log_2_ intensity >7) as high expression probes and counted the number of consecutive high expression probes per annotated gene. On alignment with the reference mouse genome, candidate fusion transcripts were scored positive if a neighboring AS L1 could be identified within 30 kb of an overlapping RefSeq gene and/or within 100 kb of the upregulated probe(s). We also required five consecutive high expression probe intensities (corresponding to adjacent exons in a given gene) in exon microarray data; length of predicted initiating genomic L1 integrant had to be >5 kb; and its subtype had to be L1 T_F_, A, G_F_ or F as per RepeatMasker (www.repeatmasker.org).

### Modified L1-reporter plasmids

To compare retrotransposition frequencies of various L1 donors, we started by replacing native L1 ORF1 sequences with a synonymous fragment of smL1 (47), by moving a *Not*I-*Xho*I fragment from pTN201 (L1spa) (50), into pBluescript-KS(+), yielding pMK20. A PstI-HindIII fragment of pMK20 was moved into pBS-KS(+), yielding pMK21. Using a QuikChange site-directed mutagenesis kit (Stratagene), we introduced a *Pac*I restriction site into the inter-ORF region in pMK21, yielding pMK22. Its 0.2-kb *Not*I-*Pac*I fragment was replaced with the 2.9-kb *Not*I-*Pac*I fragment from pCEP/smL1 (i.e. the synthetic 5′UTR and ORF1) (47), resulting in the 8.5-kb plasmid pMK22smORF1. We then subcloned its 5.6-kb *Not*I-*Hind*III fragment back into the *Not*I-*Hind*III backbone (5.4 kb) from pMK20, resulting in ∼11-kb pMK27, i.e. a marked, full-length L1 donor element. This plasmid was Sanger sequenced (Big Dye 3.1, Applied Biosystems; Transgenomic Spectrumedix), revealing a missense mutation in ORF2, i.e. Ala756Ser, along with two noncoding mutations present in pTN201. The missense mutation was repaired by replacement of a ∼1.4-kb EcoRI fragment in pMK27 with the corresponding fragment from pTN201. The 8.1-kb *Not*I-*Xho*I fragment of repaired pMK27 was ligated with a ∼11.7-kb *Not*I-*Xho*I pTN201 backbone fragment, yielding the desired final plasmid, i.e. pMK28 or L1spa::smL1-ORF1.

To preserve A/T content and synonymous amino acids of native L1s, while maximally changing codon usage, we also designed a novel recoded L1 ORF1 fragment, corresponding to 2123–2932 nt from L1spa (Supplementary Figure S2). This fragment (Blue Heron Bio), which also included 50-nt flanking arms on both ends for recombineering, was cloned into pUC MinusMCS, resulting in plasmid pJL2. The recoded L1 ORF1 fragment from pJL2 was amplified by PCR using DES3353 × DES3354 and Platinum Taq DNA polymerase High Fidelity (Invitrogen), gel purified (Qiagen), mixed with PstI-linearized pMK20 and co-transformed into electrocompetent DY380 bacteria, bearing the lambda red recombination system for recombineering (51). After heat shock at 42°C for 15 min, to induce the lambda system, bacteria were cultured on LB+Carb agar plates at 32°C overnight. Candidate clones containing recombinant pMK20: pJL2 were screened by PCR and *Pst*I digestion, and verified by sequencing. Candidate and control plasmids were digested with *Not*I and *Xho*I at 37^°^C overnight. An 8.1-kb fragment containing the synthetic ORF1 was ligated to an 11.7-kb *Not*I-*Xho*I fragment from the pTN201 backbone. The final construct, pJL3 or L1spa::recoded-L1-ORF1, was verified by Sanger sequencing.

Various AS L1 transcript overexpression plasmids were engineered from L1 T_F_ template fragments generated by PCR using HiFi platinum Taq (Invitrogen), using primer pairs DES2880 × DES2881 (L1spa nucleotides 2150–1286); DES2880 × DES2882 (nucleotides 2150–1636) and DES2879 × DES2881 (nucleotides 2823–1286). Resulting PCR products were digested with *Not*I and *BamH*I, electrophoresed on agarose gels, purified and ligated to linearized pCEP4 backbone. Fragments from AS synthetic L1 elements similarly were generated using primers DES3818 × DES3820 (amplicon mapped to corresponding coordinates in L1spa, nt 2150–1121), DES3819 × DES3820 (nt 2150–1801), DES3818 × DES3821 (nt 2823–1121) and DES3819 × DES3821 (nt 2823–1801). Products were digested with *Nhe*I and *BamH*I and ligated to similarly linearized pCEP4.

### L1 retrotransposition assays

HeLa cells were cultured in DMEM with 10% heat-inactivated fetal bovine serum and 2% penicillin/streptomycin (Gibco). Cells at ∼75% confluence in six-well plates or T25 flasks were transfected with plasmid DNA mixed with FuGENE HD (Roche) at a ratio of 1 µg to 3 µl FuGENE. To quantify transfection efficiency, GFP expression was assessed by fluorescence microscopy in cells transfected with pEGFP-N1 (Clontech). Both stable and transient transfection assays were performed. In the former (13,23), cells were treated with 0.2 mg/ml Hygromycin for various periods, starting 3 days after transfection. Hygromycin-resistant (Hygro^R^) cells then were grown without selection for several days, prior to selection for Neo^R^ L1 integrants in 0.4 mg/ml G418 (Invitrogen) for 2 weeks. In the transient assay (52), Neo^R^ L1 integrants were selected directly. After discrete colonies formed in either assay, cells were washed in 1 x phosphate buffered saline (PBS), fixed in 2% formaldehyde/0.2% glutaraldehyde in 1 x PBS, washed and stained using 0.4% Giemsa (Sigma) at RT overnight and then counted.

To assess effects of AS L1 transcripts overexpressed *in trans*, we co-transfected HeLa cells with smL1 donor plasmid together with AS smL1 fragment-expressing constructs. One µg of pCEP4/smL1/Neo donor plasmid DNA was mixed with 1 µg of various smL1 AS fragment-expressing constructs or empty vector pCEP4, respectively, in FuGENE 6. Two µg of vector pCEP4 was used as another negative control. A transient assay (52) was performed to test impacts of the AS smL1 fragments on retrotransposition, by plating cells at various dilutions and counting resulting NeoR colonies. A similar experiment was performed to assess inhibition of L1spa retrotransposition (from donor plasmid pTN201) on expression of AS L1 transcripts (see Supplementary Information).

In an independent assay of expression levels from various L1-*TEM1* reporter constructs, we conducted qRT-PCR. Plasmids were transfected into HeLa cells, and total RNAs were isolated and treated with DNase (Ambion). First strand cDNAs were synthesized using an oligo-d(T) primer. As a control, samples were treated with no reverse transcriptase. L1 expression was measured by SYBR green PCR master mix (Applied Biosystems) using ORF2 primers DES2784 × DES2790 for pTN201/TEM1, pJL3/TEM1 and pMK28/TEM1; and DES1847 × DES1848 for pCEP4/smL1/TEM1. As a control for transfection, Hygromycin gene expression was measured by qRT-PCR using DES1249 × DES1250.

### Role of Dicer in regulating L1 retrotransposition

To assay L1 retrotransposition in Dicer ex5 −/− HCT116 human colorectal cells, which are constitutively Neo^R^ (53), we used L1 donors marked by *TEM-1-*artificial intron (AI) (23). The *TEM1*-artificial intron (AI) reporter cassette and a portion of pCEP4 backbone were excised from pDES46 (which contains human L1.3) by digestion with *Not*I and *BstZ17*I. The resulting ∼13-kb backbone fragment was gel-purified. Native, hybrid or fully synthetic mouse L1 constructs in pTN201, pJL3, pMK28 and pCEP4/smL1 were digested by *BamH*I, ends were filled in by Klenow and digested by *Not*I. Each of the resulting ∼6.5-kb L1 fragments was ligated with the *Not*I - *BstZ17*I fragment. Positive candidates were confirmed by conventional Sanger sequencing. Resulting L1 donor plasmids, marked by the *TEM1*-AI reporter, included pTN201/TEM1, pJL3/TEM1, pMK28/TEM1 and pCEP4/smL1/TEM1.

To compare retrotransposition with native or hybrid L1 donors marked with *TEM1*-AI reporter in HCT116 wild-type versus Dicer −/− cell lines, transfectants were selected for 10 d on 400 mcg/ml hygromycin. Expression levels of spliced *TEM1* transcripts were assayed by qRT-PCR using DES3062 × DES3063.

## RESULTS

### Mapping an active AS promoter in mouse L1 ORF1

Previously, we and others identified mouse AS L1 RIFTs (4,45). Based on their approximate 5′ ends and widespread expression, we hypothesized that an active initiating AS promoter could reside in an AS orientation within ORF1 of mouse L1. To characterize this putative promoter experimentally, we engineered 36 candidate promoter fragments directionally upstream of a *TEM1* β-lactamase reporter gene otherwise lacking a promoter (48). To assay promoter activities of these fragments, we transfected resulting constructs individually into cultured mouse or human cells (Figure 1 and Supplementary Figure S1). The candidates were derived from mouse L1 subfamilies T_F_, G_F_, A and FIII; fully synthetic synonymously recoded smL1 (more recently called ORFeus) (47); and a novel synonymously recoded ORF1 template that we generated with A/T content similar to native elements. As positive controls, a constitutively active SV40 promoter and arrays of sense strand L1 5′ UTR monomers from T_F_ and G_F_ elements were engineered upstream of *TEM1*. As a negative control, no fragment was inserted upstream. Promoter strength scores were assigned to each fragment, based on β-lactamase reporter enzymatic activity expression, or *TEM1* transcript levels (Figure 1 and Supplementary Figure S1).

The highest level of AS promoter activity was found in L1T_F_ AS nucleotides 2823–2125, mapped as per L1spa coordinates (Figure 1A). Various L1 subfamily members displayed distinct AS promoter activities, i.e. T_F_ (∼40% of positive control, i.e. L1 T_F_ 5′ UTR monomers in sense orientation) >> G_F_ ∼ A (∼10% of control) > F (∼5% of control). For these functional promoter assays, we chose particular elements to represent the subfamilies, i.e. L1spa for L1 T_F_ subfamily; L1 G_F_62 for the G_F_ subfamily and L1Md_A2 for the A subfamily (Supplementary Figure S2). Within ORF1, these individual surrogates were 99.8, 99.7 and 99.9% identical to the consensus subfamily sequences, respectively (Supplementary Figure S2). Differences between the subfamily consensus sequences and the individual surrogates were predicted at 1944A>G and 2261G>C (i.e., L1_T_F_>L1spa, coordinates of sense strand, L1 T_F_ reference element nucleotide listed first); 1963C>T, 2687T>A, 2716T>C and 2857A>C (L1_G_F_>L1 G_F_62); and 2857G>A (L1_A>L1Md_A2).

A qRT-PCR assay for reporter transcript expression confirmed that L1 T_F_ AS promoter activity was robust, i.e. again, approximately half that of the L1 5′ UTR sense promoter (Figure 1). Low but detectable promoter activities were observed in older L1 subfamilies including F, F_II_ and/or F_III_ (Supplementary Figure S1). By contrast, virtually no promoter activity was detected in various fragments derived from the sense (coding) orientation of ORF1, AS ORF2, L1 3′ UTR, smL1 or a novel recoded ORF1 sequence which we designed to contain A/T content comparable with natural L1 sequences (Figure 1 and Supplementary Figure S1) (47).

We examined a potential basis for the broad range of AS promoter activities among different mouse L1 subfamilies. Although they are defined mainly by differences between 5′ UTR sequences, their sequences within ORF1 also are distinct (Supplementary Figure S2). Comparison of representative L1 subfamily amino acid sequences encoded by ORF1 indicated that the particular portions comprising the AS promoter were more conserved, but still distinct, between subfamilies, compared with the flanking, proximal and distal portions of ORF1 (Supplementary Figure S2). By contrast, the L1 subfamily sequences within ORF2, which do not contain this AS promoter, were nearly identical (not shown). These results suggested that ORF2 and the AS promoter segment within ORF1 may have undergone strong purifying selection (Supplementary Figure S2). A recent analysis of the evolution of mouse and human L1s confirmed that the mouse ORF1 coiled-coil domain has undergone much less adaptive change than that of human elements (15).

### RNA polymerase II transcribes AS L1 fusion transcripts

**Figure 2.**
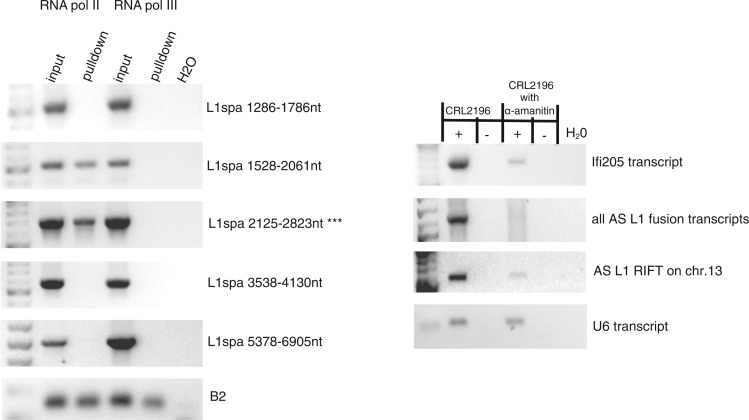
RNA polymerase II transcribes AS L1 fusion transcripts (**A**) Chromatin immunoprecipitation (ChIP) with anti-RNA polymerase II (left) and anti-RNA polymerase III (right) antibodies, followed by PCR amplification of target L1 or SINE B2 genomic sequences as indicated (right), showed specific enrichment (pulldown) of pol II at the AS L1 promoter in mouse testis (asterisks, L1 ORF1 sequences). Coordinates from L1spa reference are shown (right, cf. Figure 1A). RNA pol II also immunoprecipitated proximal L1 sequences, i.e. templates for transcribed AS fusion transcripts. As a control, both pol II and pol III pulled down SINE B2 elements genome-wide (bottom) as expected (54). (**B**) Mouse spermatocytes were treated with α-amanitin (RNA polII inhibitor) as indicated (top). Total RNAs were isolated, and reverse transcriptase was added as indicated (+ or −; top) before PCR amplification of various cDNAs as indicated (right). As a negative control, U6 transcripts (RNA pol III, not inhibited by α-amanitin) were amplified (bottom).

To confirm localization of AS promoter activity to mouse L1 ORF1 sequences and to define the RNA polymerase responsible for transcriptional initiation from it, we immunoprecipitated both RNA polymerases (pol) II and III, either of which plausibly could bind to and initiate fusion transcription from various endogenous TE sequences. As shown in Figure 2A, RNA pol II localized specifically to the ORF1 fragment that contains AS promoter activity, i.e. nucleotides 2125–2823 (Figure 1). Notably, ChIP-PCR also demonstrated that RNA pol II bound to ORF1 nucleotides 1528–2061, mapping to L1 template sequences, downstream of the AS promoter, that are expressed as AS L1 fusion transcripts. As a control, ChIP-PCR analysis of SINE B2 sequences confirmed that both RNA pol II and RNA pol III bound to those sequences (54).

To confirm the role for RNA pol II in transcribing AS L1 RIFTs, we treated a mouse spermatocyte cell line, CRL2196, with α-amanitin (Figure 2B), a potent and specific RNA pol II inhibitor. We assayed AS L1 RIFT expression by qRT-PCR, demonstrating substantial inhibition by this drug both in general and at individual loci. Together with ChIP-PCR, our results indicated that AS L1 RIFTs were transcribed by RNA pol II.

### Identification of diverse AS L1 RIFTs

To find mouse transcripts that included sequences from genomic L1 templates, we screened full-length transcripts represented in bacteriophage libraries. Although 6–30% of all mouse and human transcripts recently were reported to be initiated from TEs including L1s (17), we observed that only ∼0.1 and 0.03% of all transcripts in phage cDNA libraries representing testis and thymus, respectively, hybridized with an L1 T_F_ subfamily probe for ORF2 (hereafter called ORF2^+^ transcripts, Figure 1 and Supplementary Table S1B). Sequential hybridization with an L1 T_F_ ORF1 probe (Figure 1) revealed an additional 0.06% of testis transcripts and 0.02% of thymus transcripts, identifying those that contained 5′ L1 ORF1 but not ORF2 sequences. Of 940 testis cDNA clones hybridizing with either probe, 363 (∼39%) were ORF1^+^. Similarly, of 253 thymus cDNA clones hybridizing with either probe, 99 (∼39%) were ORF1^+^.

We hypothesized that such ORF1^+^ORF2^-^ transcripts would include AS L1 RIFTs. This possibility was prompted by our previous identification of fusion transcripts in adult mouse tissues, mapping to L1 elements (4). Of 27 ORF1^+^ORF2^-^ transcripts identified from testis, 21 (78%, Supplementary Table S2) contained AS L1 ORF1 sequences spliced with other exons in the sense orientation, forming AS L1 RIFTs. Additionally, 2 of 13 thymus cDNAs (15%, Supplementary Table S2) also were spliced AS L1 RIFTs. Other ORF1^+^ORF2^-^ cDNAs either were unspliced AS RIFTs, reading antiparallel to ORF1 through the 5′ UTR into flanking genomic sequences (4 in testis, 15% of total; 2 in thymus, 15%), or were prematurely polyadenylated, sense-strand transcripts (2 in testis, 7%; 9 in thymus, 69%) (55). Some RIFTs were initiated in other mouse strains by polymorphic L1s absent from the C57BL/6 J (B6) reference genome (4,5). These screens also showed that some AS RIFTs were readily detectable without PCR amplification.

We identified diverse spliced ORF1^+^ORF2^-^ transcripts initiated across the genome in a variety of chromosomal and tissue contexts, as illustrated by schematics of their genomic templates including the initiating L1 elements (Supplementary Figure S3) (56). To determine whether AS L1 RIFTs were expressed more broadly, we screened additional mouse strains and cell lines by qRT-PCR. We experimentally identified 41 additional AS L1 RIFTs (Supplementary Table S2) expressed in cultured mouse spermatocyte cells or adult testes. Twelve (29%) aligned to genomic regions lacking a previously annotated gene, and two (5%) were initiated from polymorphic L1s absent from B6 mice (5). In addition, we searched public expressed sequence tag (EST) libraries by BLAST alignments (45), revealing 15 additional full-length mouse testis ESTs (57) as spliced AS L1 RIFTs (Supplementary Table S2). Fifty-seven EST clones contained AS L1 sequences in their 5′ ends. Of these, 22 were spliced, but no splicing was observed within L1 sequences *per se*. Many of the AS L1 RIFTs identified by bioinformatics analysis were found in testis and embryonic cells at certain developmental stages, again suggesting a high level of tissue specificity. This search identified >80 EST clones with AS alignment ≥300 nt and >90% identity with L1 at their 5′ ends, of which 15 were full-length RIKEN cDNAs. In some cases, 3′ paired ends of other EST clones were identified from the EMBL/EBI database using 5′ clone IDs; 57 clones were sequenced from both ends.

To compare RIFT expression levels in different tissues, we re-assayed 17 RIFTs identified initially in adult testis or from a spermatocyte cell line (Supplementary Figure S4). As expected, almost all of these RIFTs were confirmed in testis. Relatively few were expressed in other tissues assayed, but we did recover clones 1ASII1, additionally expressed in 11-day embryos; L1-5AS1-1, additionally expressed in brain; and CRL2196C10, widely expressed in most tissues assayed. We also assayed for overlapping spliced transcripts from cognate genes. Although AS L1 RIFTs that were spliced to downstream exons of *Erbb2ip*, *Usp29* and *Arhgap15* each were expressed in testis, the corresponding conventional transcripts of these genes (i.e. lacking sequences from L1s) were not detectable there.

To identify genes whose expression levels may be affected by AS L1 RIFTs, we probed Affymetrix mouse exon microarrays conventionally with total RNAs. As commercial exon microarrays typically exclude probes for repetitive elements such as L1 retrotransposons, we also developed a novel, unconventional assay using the arrays to screen specifically for AS L1 RIFTs that include downstream exons. In this assay, hereafter called the RIFT assay, we prepared cDNAs from several tissues and mouse lineages by RT-PCR, using an AS L1-specific primer paired with an oligo-d(T) primer. At least 130 unique spliced AS L1 RIFTs were identified in adult testis (Supplementary Table S2), of which many were also identified in phage cDNA libraries (Supplementary Table S2). Thus, many transcripts were corroborated by independent methods.

**Figure 3.**
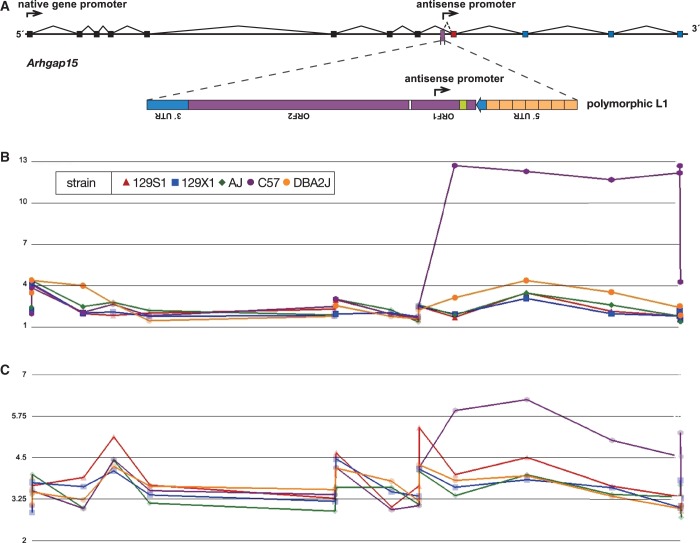
Contribution of an AS L1 RIFT to overall *Arhgap15* gene expression in various mouse strains. (**A**) Schematic representation of *Arhgap15* exons, including a polymorphic AS L1 integrant in the B6 reference genome but not in other strains. (**B**) AS L1 RIFT expression at *Arhgap15* was detected in B6 mice, using the novel RIFT assay where we performed RT-PCR using AS L1 and oligo-d(T) primers, followed by hybridization of resulting cDNA products to an Affymetrix mouse exon microarray. We required five consecutive exon probes to be strongly positive to call RIFTs. Shown are genomic positions of probes within exons (x-axis) and hybridization signal intensities on a log scale (y-axis). *Legend, inset*: five mouse strains, different symbol colors and shapes. (**C**) Conventional assay for *Arhgap15* expression in total RNAs (see legend, B). The AS L1 RIFT in B6 mice affects total RNA expression levels at the 3′ exons downstream of the polymorphic, initiating L1 integrant (see corresponding positions, A).

Both assays, i.e. the RIFT assay and conventional expression profiling using exon microarrays, confirmed the expression of an AS L1 RIFT at *Arhgap15*, initially found by screening a testis cDNA library (4). The initiating L1 integrant is polymorphic (5) and is oriented antiparallel to the transcription unit of *Arhgap15*. The AS L1 RIFT, expressed in the same orientation as the overlapping gene’s reading frame (Figure 3), was readily detectable in RNA from B6 but not the other strains tested, consistent with the presence or absence of the initiating L1 element. Both assays showed that this AS RIFT contributed to overall *Arhgap15* RNA levels, in particular those measured at its 3′ end*.*

**Figure 4.**
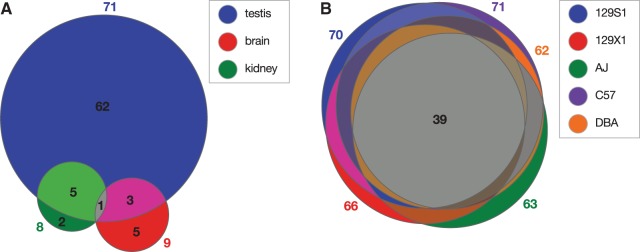
Comparison of AS L1 RIFTs expressed in various mouse tissues and strains. Distinct AS L1 RIFTs were counted in Venn diagrams depicting shared (*overlapping) and unique (*distinct) RIFTs expressed in different mouse strains and tissues. Numbers indicate unique RIFTs in each group. (**A**) AS L1 RIFTs expressed in B6 testis (n = 71, blue), brain (n = 9, red) and kidney (n = 8, green); (**B**) AS L1 RIFTs expressed in testis of five mouse strains: 129S1 (n = 70, blue), 129X1 (n = 66, red), A/J (n = 63, green), B6 (n = 71, purple) and DBA/2 J (n = 62, orange).

The RIFT assay also showed that distinct AS L1 RIFTs, although expressed in various tissues, were most abundantly expressed in testis. Several other RIFTs were identified in brain and kidney (Figure 4). Notably, a few RIFTs were expressed in more than one tissue. Thus most, but not all, RIFTs were expressed in a tissue-specific fashion. In addition, comparison of RIFTs expressed in five diverse strains highlighted that approximately half were conserved in all five strains (Figure 4), implying that potential biological functions of some RIFTs may be shared. Other RIFTs were expressed only in particular strains, consistent with the presence of the polymorphic L1 AS promoter in about half of these cases and with differential RIFT expression in the others.

Using targeted RT-PCR, we observed ∼40% of extant L1 T_F_ subfamily members studied here initiated a nearby AS L1 RIFT. About 13% of L1 G_F_ elements, about 4% of A elements, and zero of one F element initiated RIFTs (4). Overall, about 19% of 68 genomic elements initiated RIFTs (data not shown).

### AS L1 RIFT TSS are proximal to the AS L1 promoter

**Figure 5.**
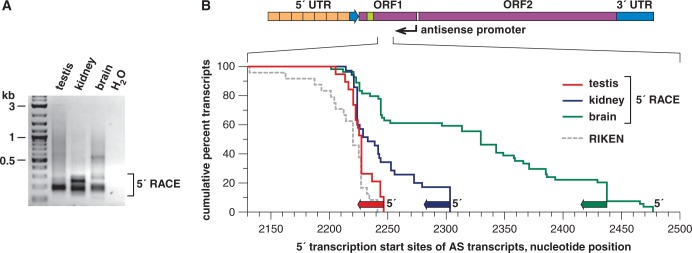
AS transcription start sites found by 5′ RACE in multiple tissues. (**A**) A 5′ RACE was performed by PCR for 5′ ends of AS L1 RIFTs, using total RNAs from testis, kidney and brain. Products were separated by agarose gel electrophoresis. Individual cloned 5′ ends were sequenced from these pools. (**B**) The cumulative positions of TSS for AS L1-gene RIFTs are plotted by summing individual transcripts’ 5′ ends, mapped against coordinates from L1spa. We analyzed 19 5′ RACE clones from testis (red), 35 from kidney (blue) and 54 from brain (green). Also superimposed here are the cumulative positions of 5′ ends from 24 RIKEN clones that align well with L1spa, although these formally are not ends determined by 5′ RACE cloning (Supplementary Table S2) (45).

To identify the 5′ TSS of AS L1 RIFTs, we performed 5′ rapid amplification of cDNA ends (5′ RACE) analysis on fusion transcripts expressed in testis, kidney and brain. A primer specific for the L1 T_F_ ORF1 template was paired with a standard RACE primer for amplification from total RNAs. A range of PCR product sizes was observed, revealing multiple nearby TSS (Figure 5). A large fraction of the 5′ ends of transcripts recovered from all three tissues mapped to ORF1 nucleotides 2201–2244. In kidney and brain, additional TSS mapped to a wider range of ORF1 sequences, i.e. nucleotides 2210–2306 and nucleotides 2210–2478, respectively. These results correlated well with the 5′ ends of RIFT cDNAs identified in phage libraries (Supplementary Table S2). In addition, the 5′ ends of 24 RIKEN cDNA clones, most of which were reported previously (45), mapped to this same region. Thus, the 5′ TSS of the fusion transcripts, determined experimentally by 5′ RACE analysis and from cDNA clones, were closely adjacent to the experimentally mapped AS L1 promoter (Figure 1).

We observed a candidate transcript-initiating TATAA sequence at position 2698 of AS L1 T_F_ ORF1 (Supplementary Figure S2), but it is likely too distant from the RIFTs’ 5′ ends, identified by RACE (Figure 5), to account for them. Nevertheless, many mouse and human promoters lacking TATAA sequences have been identified previously, including variants of an ‘initiator element (Inr)’ sequence (58). We noted several variants of this sequence within the mapped AS promoter, some of which were immediately adjacent to observed TSS in the RIFTs.

### Other predicted features of AS L1 RIFTs

To determine whether there are canonical splice donor sites and predicted translation start sites in the L1 templates for AS RIFTs, first we mapped an arbitrary collection of 65 spliced, fully sequenced AS L1 RIFTs to the reference genome. The cDNAs were spliced mostly at one of two consensus donor sites. The most common donor, used in 44 (68%) of 65 RIFTs, was GATGgtgag (coordinate 1838 of L1spa, Figure 1). Another common splice donor in 13 (20%) transcripts was TCAGgtgtg (L1spa coordinate 1892). Both of these donor sites included conventional splicing sequences. Conceptual translation of fusion transcripts revealed that eight predicted translation start sites (ATG) occurred within the AS ORF1 sequences of the RIFTs, in at least two of three possible reading frames in a variety of sequence contexts, suggesting that fusion proteins may be expressed from many diverse transcripts.

### Effect of genomic context on AS L1 RIFT expression

To assess whether variable position effects or gene-specific expression differences (16) could influence AS promoter activity differentially at distinct chromosomal loci, we asked whether comparable intronic AS promoters, located in various genomic locations but present within the same tissues, could have similar activities. We identified 13 (19%) of 68 polymorphic full-length L1s, which initiated AS L1 RIFTs in testis, as assayed by RT-PCR (4). Thus although a significant fraction of distinct L1 AS promoters initiated AS L1 RIFTs, a majority did not, even in ‘transcriptionally capable’ tissues such as testis. This observation suggests that the genomic contexts (16) of comparable extant L1 integrants can influence their expression of AS RIFTs*.*

### Impacts of AS transcription on L1 transcription and retrotransposition

The synthetic mouse L1 element smL1 retrotransposes ∼200-fold more than endogenous mouse L1s (47). Increased RNA polymerase II processivity and increased expression of L1 ORF1 and ORF2 were proposed to be causes of this increase (47). Compared with mouse smL1, a synthetic human L1 (ORFeus-Hs) retrotransposed only about 3-fold more than the most active native human L1 elements (59). The exact basis for the differential increase in retrotransposition by synthetic mouse more than synthetic human L1s, over the corresponding native elements, is unknown. We noted that smL1 lacked the AS promoter activity harbored in ORF1 by native mouse L1s (Figure 1), thereby plausibly contributing to marked increases in its expression and retrotransposition. To test this possibility, we replaced native ORF1 in L1spa with the synonymous fragment from smL1, forming a novel, hybrid full-length L1 donor. To assess the role for A/T content in affecting L1 transcript levels, we also synthesized a second partially recoded hybrid L1 donor, i.e. as in pJL3. Like smL1, the recoded L1 in pJL3 also lacked AS promoter activity (Figure 1), but it had higher A/T content, similar to that of native mouse L1 elements.

We also measured transcript levels expressed from these native or hybrid L1 donor elements using qRT-PCR. The lowest L1 transcript levels were observed for native L1 T_F_ (L1spa), whereas the highest levels were seen for full-length smL1 (Supplementary Figure S6). Intermediate levels were seen for the novel hybrid element containing recoded ORF1, harboring no AS promoter activity and neutral changes in A/T content, engineered upstream of native L1spa (T_F_) ORF2. Somewhat higher expression was seen for the second hybrid L1 element, i.e. smL1/L1spa in pMK28, which has lower A/T content in ORF1 (47). The results suggested a potential contribution by native AS L1 promoters in reducing L1 transcription.

**Figure 6.**
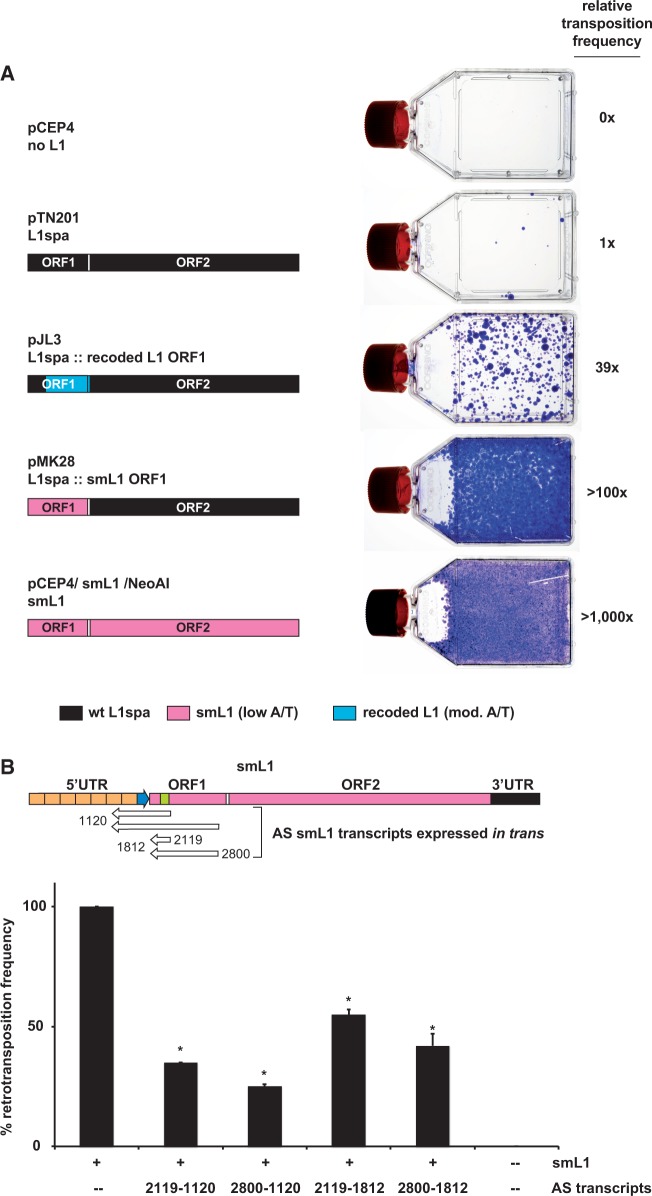
AS L1 transcription helps to limit retrotransposition. (**A**) *Cis* effects. Native L1 ORF1 sequences in L1spa (*black*) were replaced either with a synonymously recoded fragment from smL1 with its markedly reduced A/T content (47) (pink), or a new recoded fragment that preserves A/T content more similar to that found in endogenous L1s (blue). Resulting marked L1 donors, i.e. pJL3 and pMK28, were assayed for retrotransposition by transfecting human HeLa cells. As controls, native L1spa (in pTN201) (50), smL1 (in the same pCEP4 donor plasmid backbone and marked with Neo^R^/AI) and an empty donor plasmid (pCEP4) were transfected in parallel. Following selection on hygromycin for 47 d, 1 million Hygro^R^ cells were plated per flask, and new L1 integrants were selected for Neo^R^, followed by staining of colonies. Retrotransposition frequencies are indicated relative to L1spa in pTN201 (right). (**B**) *Trans* effects. To measure overexpressed AS smL1 RIFTs' suppressive effects on retrotransposition by smL1, first we directionally cloned four AS fragments from smL1, i.e. coordinates 2119-1120 (PCR amplicons DES3820 × DES3818, Supplementary Table S1); 2800–1120 (DES3821 × DES3818); 2119–1812 (DES3820 × DES3819); and 2800–1812 (DES3821 × DES3819) into pCEP4 downstream of its strong CMV promoter. Each cloned construct was co-transfected into HeLa cells with the smL1 retrotransposition donor plasmid, pCEP4/smL1/Neo. As positive and negative controls, smL1 donor alone and pCEP4 alone were transfected into HeLa cells, respectively. After transfection, cells were plated at various dilutions, selected on G418 for 2 weeks and Neo^R^ colonies were stained and counted (see Supplementary Figure S7). The mean and range of duplicate counts were determined, and retrotransposition frequencies were normalized relative to that of the smL1 positive control (defined as 100%). Asterisks: significantly different from control retrotransposition frequency (two-tailed *t*-test, *P* < 0.05 in all pairwise comparisons).

We also compared mobilization of the various engineered L1s (Figure 6) (13). The hybrid L1 with reduced ORF1 A/T content in pMK28 retrotransposed at least 100-fold more than native L1spa (Figure 6). The partially recoded hybrid L1 in pJL3, with neutral changes in ORF1 A/T content, mobilized up to ∼39-fold more than native L1spa. We conclude that synonymous disruption of the AS L1 promoter in ORF1, regardless of its A/T content, can increase retrotransposition substantially. These results are also consistent with evidence showing that longer L1 templates bearing reduced A/T content can result in increased transcript levels and retrotransposition (47). Thus, the AS L1 promoter helps to limit retrotransposition in *cis*.

To determine whether overexpressed AS L1 transcripts could inhibit retrotransposition in *trans*, first we engineered AS smL1 fragments to overexpress them in the desired orientation. Four AS fragments from smL1, corresponding to AS L1spa coordinates 2119–1120, 2800–1120, 2119–1812 and 2800–1812, each were cloned downstream of the CMV promoter and were co-transfected with marked smL1 in a transient retrotransposition assay (52) (Figure 6 and Supplementary Figure S7A). As a positive control, where smL1 could mobilize in the absence of overexpressed AS L1 transcripts in *trans*, empty pCEP4 was co-transfected with smL1. A negative control consisted of cells transfected with no smL1 donor and pCEP4 alone. The overexpression of AS smL1 transcripts in *trans* significantly suppressed smL1 retrotransposition*,* i.e. by ∼50–75% (Figure 6). This level of repression was comparable with that of human L1 siRNAs (34). In another experiment, several distinct native AS L1T_F_ transcripts (generated from L1spa template at coordinates 2823–1286, 2150–1286 and 2150–1636; cf. Figures 1 and 5, Supplementary Figure S3 and Supplementary Table S2) were overexpressed (Supplementary Figure S7). These AS L1 transcripts overlapped in part with endogenous AS L1 RIFTs (Figure 1). Their expression in *trans *suppressed L1 retrotransposition at comparable levels, i.e. 2 - to 5-fold (Supplementary Figure S7B).

### Modest role of Dicer in limiting native L1 retrotransposition

We hypothesized that AS transcripts initiated from the AS promoter, expressed together with sense transcripts initiated from the conventional 5′ promoter of mouse L1s, could result in the formation of double-stranded (ds) RNAs. In turn, these dsRNAs could trigger formation of short interfering RNAs or microRNAs through a Dicer-dependent pathway (60,61), thereby reducing sense strand L1 transcripts and limiting retrotransposition. We tested this possibility by using Dicer knockout cells in a retrotransposition assay (23). Because Dicer ex5 -/- HCT116 human colorectal cells are Neo^R^ (53), we engineered novel L1 donors, marked with the β-lactamase *TEM1* reporter interrupted by an artificial intron (13). Either native or hybrid recoded L1s were transfected into HCT116 Dicer ex5 −/− cells and control wild-type Dicer cells. After selection on donor plasmids, retrotransposition was assayed by qRT-PCR analysis of spliced *TEM1* transcripts, expressed from new L1 insertions (62). The retrotransposition rate of L1spa, which contains an active AS promoter, increased slightly, i.e. <2-fold, in Dicer−/− cells compared with control cells. By contrast, retrotransposition by recoded elements lacking AS promoter activity, i.e. pJL3/TEM1, pMK28/TEM1 and pCEP4/smL1/TEM1, was essentially unchanged in Dicer−/− cells versus control cells (Supplementary Figure S8). Thus, Dicer played a modest role in suppressing native L1 retrotransposition, mediated by AS L1 transcription; most of the suppression by AS L1 transcripts occurred independent of Dicer. Previous experiments showed a similar ∼2-fold level of suppression of human L1 retrotransposition on knockdown of Dicer in cultured cells. That result was interpreted as showing the role for Dicer-dependent RNA interference in regulating human retrotransposition (34).

## DISCUSSION

A recent analysis of human and mouse transcriptomes suggested that 6–30% of all transcripts are initiated from repetitive elements (17). Here, we have identified and experimentally characterized an active initiator of such transcripts, i.e. an AS promoter within ORF1 of mouse L1 retrotransposons, present in thousands of full-length copies genome-wide, more than its human counterpart (4,23). It initiated a diverse range of fusion transcripts, as shown by >100 distinct AS L1 RIFTs identified here and elsewhere (Figure 3, Supplementary Figure S3 and Supplementary Table S3) (4,45). AS L1 RIFTs included spliced, unspliced and/or noncoding RNAs, and were readily detected in various mouse cell lines, tissues, developmental stages and strains (Figure 4 and Supplementary Table S2) (4). In addition to adding significantly to transcriptional diversity, AS L1 transcription helped to limit L1 retrotransposition (Figure 6, Supplementary Figures. S6 and S7).

### Characterization of an AS L1 promoter and AS L1 RIFTs

The co-existence of a protein-coding sequence together with an antiparallel promoter activity in opposite overlapping orientations is unusual, but is not unprecedented, in mammalian genomes (63–65).

Many sequence differences, particularly in the 5′ UTR and within ORF1, distinguished the three active mouse L1 subfamilies, *i.e.* T_F_, G_F_ and A elements (Supplementary Figure S2). Several putative transcription factor binding sites in the AS promoter sequence of L1spa (50) and other T_F_ subfamily elements could be altered by natural sequence variants occurring in other L1 subfamilies (Supplementary Figure S2). Although members of each subfamily retrotransposed recently (4,14,50,66), these sequence differences simultaneously could affect both their distinct retrotransposition rates, by affecting ORF1p structures, and their AS promoter activities. We note that a single amino acid substitution in mouse ORF1p can affect L1 retrotransposition (67). In addition, the recoded synonymous sequences in ORF1 of pMK28 and pJL3 disrupted numerous predicted transcription factor binding sites in the AS promoter (Supplementary Figure S2), consistent with a complete lack of AS promoter activity observed in those elements (Figure 1).

The various AS promoter activities associated with each L1 subfamily (Figure 1) were roughly proportional to the number of RIFTs initiated by them *in vivo* (Supplementary Table S2 and Supplementary Figure S3). Thus, we concluded that AS L1 promoter activities ranked as L1 T_F_ >> G_F_ ∼ A > F. Notably, the latter subfamilies possessed modest, but detectable, AS promoter activities.

Estimated ages, counts and retrotransposition frequencies of L1 subfamily members have varied considerably. The average ages of L1 T_F_ elements range from 0.25 to 1.23 million years old (15), and numbers of full-length insertions range from 3400 (4,68) to ∼4800 (15), whereas active and/or polymorphic TF elements ranged from ∼1900 (4) to 3000 (68). The average ages of L1 G_F_ subfamily members have been estimated at 0.75 to 2.16 million years (15). Full-length G_F_ element counts have varied from 704 (4) to 1 500 (14). There are ∼400 (14) to 535 (4) active and/or polymorphic L1 G_F_ elements. The average ages of the youngest L1 A subfamily members have been estimated to range from 0.21 to 2.15 million years, and older A subfamilies have also been identified (15). Full-length A elements have ranged in number from 3400 (15) to 6500 (66). There are ∼900 (14) to 1600 (4) active and/or polymorphic L1 A insertions. Individual elements of all three subfamilies have been shown to retrotranspose at comparable frequencies (14).

These findings prompted us to consider an apparent paradox. How might T_F_ subfamily elements harbor the strongest AS promoter activity, even though they have accumulated to some of the highest copy numbers of full-length L1 integrants in the genome (4,15)? We speculate that more robust host defenses might be necessitated by elements with increased retrotransposition potential, thereby resulting in relatively equivalent mobilization frequencies of distinct subfamily elements (14). This paradox could also be explained by comparing the long evolutionary times over which different subfamilies have accumulated, moving in germ line tissues under negative selection (15), versus the expression of AS L1 RIFTs in germ line and somatic tissues, measured in real time.

Although we detected both sense and AS L1 transcripts expressed in the same tissues, including testis and thymus (cf. Supplementary Table S2), in this study we have not tested whether sense and AS L1 promoters may be active simultaneously in single cells. If they are not, the resulting unbalanced expression of sense versus AS L1 transcripts in distinct cells or tissues could allow particular L1 elements to evade this putative defense mechanism. Moreover, individual mouse and human L1 elements can mobilize over a wide range of frequencies, despite similar ORF sequences shared by ‘hot’ versus ‘cool’ elements (14,69–71). Although we found many diverse AS L1 RIFTs expressed, many were expressed at low levels, and many other potentially active, distinct L1 elements had no detectable AS RIFT expression.

We used several independent experimental methods to identify AS L1 RIFTs (Figure 3 and Supplementary Table S2). These included screens of phage cDNA libraries, RT-PCR followed by cloning and sequencing, bioinformatics surveys of transcript sequence databases, Northern blots (4) and a novel RIFT assay using RT-PCR followed by exon microarray hybridization. Considered together with results from 5′ RACE analysis (Figure 5) and *in vitro* promoter assays (Figure 1), these findings clearly established that many diverse RIFTs were expressed from AS promoters located in L1 ORF1 *in vivo*.

Many additional AS L1 RIFTs might have been missed in our study, owing to a lack of saturation of our screens; a limited range of mouse tissues and lineages used in the various screens; low expression levels; and/or strict criteria imposed in our RIFT assay. Even so, after summing up all AS L1 RIFTs observed by various methods, we conclude that the robust AS L1 promoter activity characterized here still does not account for most of the 6–30% of all transcripts initiated from transposons in mouse (17). A possible explanation is that other, still unidentified, promoters inside or outside of TEs initiate such abundant transcripts. We are currently working to identify such potential promoters, but, to date, no experimental evidence for them has been reported. Alternatively, this reported range (17) could dramatically overestimate actual TE-initiated transcription. Our phage library screens revealed ∼0.03 to 0.1% of all transcripts hybridized with an L1 ORF2 probe (Figure 1), far less than identified from CAGE tags (17). In addition, recent studies in mouse embryonic stem cells identified most L1-specific small RNAs mapping to both strands of the L1 5′ UTR and proximal ORF1, but not ORF2 or the 3′ UTR (43,44).

The presence of a particular full-length L1 element was necessary, but not sufficient, to initiate a locus-specific AS L1 RIFT. We found that only 13 (19%) of 68 polymorphic full-length L1s initiated AS L1 RIFTs in testis, as assayed by RT-PCR. Moreover, some RIFTs only were expressed in embryonic, newborn or adult mouse testis, whereas smaller numbers were expressed in other organs such as brain and kidney (Figure 4). A few AS L1 RIFTs were expressed in several tissues (Figure 4). We speculate that the determinants of variable initiation of RIFTs by various L1s across the genome may include position effects, neighboring transcription units, other nearby genomic features, tissue-specific factors and/or variable chromatin marks (16). Alternatively, certain L1 integrants could undergo differential, transcriptional gene silencing *in situ* (72) (Kannan,M. *et al.*, in preparation).

### Biological roles of AS L1 RIFTs

L1 retrotransposons are actively mobilized in mouse and human germ lines, resulting in substantial, ongoing structural variation in both genomes (4,5,73,74). In addition, L1s may retrotranspose in somatic tissues such as the brain, during normal development, and in certain cancers, resulting in somatic mosaicism (75–78). Because AS promoters (including many polymorphisms) are inherently part of many such integrants, they could contribute substantially to natural transcriptional variation distinguishing between lineages, individuals and even cells (4,16). In addition to the robust level of AS L1 RIFT expression at *Arhgap15* (Figure 3), we previously reported comparably robust levels of AS L1 RIFT and native transcripts at *Drosha*, as shown by northern blot (4). However, aside from these cases, most other mouse AS L1 RIFTs appear to be expressed at low levels, as in human (27). Further experiments to quantify and compare RIFT expression levels versus long noncoding RNAs (79), microRNAs and other biologically significant transcripts are warranted.

AS L1 RIFTs frequently can be expressed from nonpolymorphic L1 integrants in diverse mouse lineages (Figure 4), implying that at least some may share a conserved, albeit unknown, biological function. Certain expressed RIFTs (Supplementary Table S2) may play several distinct biological roles including possible protein translation. In some cases, the predicted protein-coding ORF sequences of AS L1 RIFTs match the cognate ORF in transcripts from the associated native genes, suggesting that although they may encode identical proteins, their expression patterns may be added to, or modified by, the AS L1 promoter. Other AS L1 RIFTs may modify or replace cognate protein structures or expression, generate novel proteins or long noncoding RNAs (25,26,46,80) or introduce different 5′ UTR sequences that could alter translational regulation. Transcripts that are AS to canonical sense transcripts could play other roles including degradation of sense strand transcripts through RNA interference or Dicer-independent mechanisms (42), variable compartmentalization and/or effects on transcript splicing and termination, RNA editing and translation (25,42,65).

We also found that AS L1 transcription also limited L1 retrotransposition, as demonstrated both by altered L1 transcript levels (Supplementary Figure S6) and mobilization on synonymous recoding of the AS L1 promoter in ORF1 in *cis* and upon overexpression of AS L1 RIFTs in *trans *(Figure 6 and Supplementary Figure S7). Hybrid L1s, containing either a recoded synonymous ORF1 segment from smL1 with decreased A/T content (47) or a second recoded ORF1 segment with neutral changes in A/T content, exhibited higher rates of retrotransposition than that of native L1spa (Figure 6). The native AS L1 promoter could inhibit L1 retrotransposition in *cis *by triggering transcriptional interference, i.e. convergent, bidirectional transcription (81). Expression of AS L1 transcripts alternatively could result in formation of double-stranded (ds) RNA molecules that could affect chromatinization and silencing of the L1 template (42) or trigger an interferon response (82). Such dsRNAs could form substrates for processing to small inhibitory RNAs through Dicer-dependent (60) or -independent mechanisms (42). Interestingly, a modest number of ∼23-nt small RNAs that map to the mouse L1 5′UTR region recently were identified in testis and in full-grown and meiosis I oocytes (83). In addition, both sense and AS small RNAs, mapping to the 5′ end of mouse L1 elements, have been identified in mouse ES cells (43,44). Thus, both human and mouse L1 retrotransposition can be inhibited by RNAi in various cellular contexts (34,43,44).

We showed that Dicer played a modest <2-fold role in suppression of endogenous mouse L1 elements (Supplementary Figure S8), the only elements capable of triggering dsRNAs that were tested here. By contrast, Dicer appeared to be a crucial component in RNAi-mediated regulation of L1 expression and mobilization in mouse ES cells (43,44). We found that retrotransposition of pJL3/TEM1 was higher than that of pTN201/TEM1, even without Dicer (Supplementary Figure S8). For this reason, we speculate that the RNAi pathway is not likely to be the predominant suppressive mechanism of mouse L1 elements, and that other suppressive mechanisms are involved, at least in the differentiated somatic cells tested here (Supplementary Figure S8). Thus, we conclude that AS L1 transcripts act mostly independent of Dicer in decreasing L1 expression and retrotransposition.

In summary, we conclude that mouse L1s encode a built-in mechanism that regulates themselves and alters expression of neighboring genes. We note a similar organization of bidirectional promoters resides in most other classes of autonomous mammalian retrotransposons, including human L1s and mouse and human LTR retrotransposons (54,60,84–86). Interestingly, bidirectional transcription at a particular mouse SINE B2 element was found to help establish an insulator or boundary element that, in turn, is critical to the developmental regulation of a neighboring gene (54). The evolutionary implications of such self-antagonizing promoters may be that transposons, including mouse L1 retrotransposons, can thereby limit their own expression. This would reduce their deleterious effects and costs to the fitness of their host (87), while exaptively modifying and diversifying the structure, expression and control of many other genes (25,84).

## ACCESSION NUMBERS

GenBank accession numbers EU233991 - EU234054 are included in tables with novel sequences.

## SUPPLEMENTARY DATA

Supplementary Data are available at NAR Online.

## FUNDING

Funded by the Intramural Research Program, Center for Cancer Research, National Cancer Institute (NIH); federal funds from the National Cancer Institute, NIH to SAIC-Frederick [contract N01-CO-12400]; and Ohio State University Comprehensive Cancer Center. Funding for open access charge: Ohio State University Comprehensive Cancer Center.

*Conflict of interest statement*. None declared.

## Supplementary Material

Supplementary Data
