# Supplementary material for: An antisense promoter in mouse L1 retrotransposon open reading frame-1 initiates expression of diverse fusion transcripts and limits retrotransposition
Source: Nucleic Acids Res. 2014 Jan 31;42(7):4546–62. doi: 10.1093/nar/gku091 (PMC3985663; doi:10.1093/nar/gku091)
Supplement: Supplementary Data [file supp_42_7_4546__index.html]

An antisense promoter in mouse L1 retrotransposon open reading frame-1 initiates expression of diverse fusion transcripts and limits retrotransposition — An antisense promoter in mouse L1 retrotransposon open reading frame-1 initiates expression of diverse fusion transcripts and limits retrotransposition — Supplementary Data 

# An antisense promoter in mouse L1 retrotransposon open reading frame-1 initiates expression of diverse fusion transcripts and limits retrotransposition

## Supplementary Data

files

**Files in this Data Supplement:**

- Supplementary Data - pdf file
