# Supplementary material for: An antisense promoter in mouse L1 retrotransposon open reading frame-1 initiates expression of diverse fusion transcripts and limits retrotransposition
Source: Nucleic Acids Res. 2014 Jan 31;42(7):4546–62. doi: 10.1093/nar/gku091 (PMC3985663; doi:10.1093/nar/gku091)
Supplement: Supplementary Data [file supp_gku091_nar-02689-v-2013-File007.pdf]

Li et al. An antisense promoter in mouse L1 retrotransposon open reading frame-1 (ORF1) initiates expression of diverse fusion transcripts and limits retrotransposition

SUPPLEMENTARY TABLES.

Table S1. **Candidate promoter fragments and primers used in this study.**

(A) Candidate mouse L1 promoter fragments, for testing in promoter assays (Fig. 1, Fig. S1), were recovered by PCR amplification using forward and reverse primers as indicated here, and cloned directionally upstream of *TEM1* beta-lactamase reporter. Restriction endonuclease sites for directional subcloning are indicated (*lowercase*).

Frag.	Subfamily	Oligo IDs	Position in L1spa	Length	Ori.	Forward oligo (5'- 3')	Reverse oligo (5'- 3')
1	T_F	1212/1213	14-1786	1773bp	s	agatctATTAGTCTGAACAGGTGAGAGG	ccatggCTGGTAATCTCTGGAGTTAG
2	T_F	1298/1797	2150-1801	350bp	as	ccatggACGGAGGAATCTTACTAACAGG	agatctGAGTTTTCTTTATTGTGTCTACTTCC
3	T_F	1298/1219	2648-1801	848bp	as	ccatggACGGAGGAATCTTACTAACAGG	agatctCTGGTGTAATTCTGATAGGCTTG
4	T_F	1299/1797	2150-1883	268bp	as	ccatggCCCAACACACCTGAGAACCT	agatctGAGTTTTCTTTATTGTGTCTACTTCC
5	T_F	1299/1219	2648-1883	766bp	as	ccatggCCCAACACACCTGAGAACCT	agatctCTGGTGTAATTCTGATAGGCTTG
6	T_F	1218/1220	2823-2125	699bp	as	ccatggGGAAGTAGACACAATAAAGAAAACTC	agatctTTCGTGGAGAGATAATGCGTG
7	T_F	1218/1221	3136-2125	1012bp	as	ccatggGGAAGTAGACACAATAAAGAAAACTC	agatctCCTTTCATTCTGAGGTAGTGTC
8	T_F	2093/1220	2823-2626	198bp	as	ccatggCAAGCCTATCAGAATTACACCAG	agatctTTCGTGGAGAGATAATGCGTG
9	T_F	2093/1221	3136-2626	511bp	as	ccatggCAAGCCTATCAGAATTACACCAG	agatctCCTTTCATTCTGAGGTAGTGTC
10	T_F	1221/1798	3136-2803	334bp	as	ccatggCACGCATTATCTCTCCACGAA	agatctCCTTTCATTCTGAGGTAGTGTC
11	T_F	1459/1460	4420-2931	1490bp	as	ccatggCACAAGAACAGAATGCCACC	agatctTCTTTGAAGGTCTGATAG
12	T_F	1461/1462	5296-4471	826bp	as	ccatggGTATTCTACCCAACTCATTTTATG	agatctTTCACTTCCTTCGTTAG
13	T_F	1463/1464	6905-5378	1528bp	as	ccatggATGGATTGGCAGGACCAAC	agatctGTGTTTTGTTCCCACTTCTAAG
14	T_F	2096/2097	1801-2150	350bp	s	agatctACGGAGGAATCTTACTAACAGG	ccatggGAGTTTTCTTTATTGTGTCTACTTCC
15	T_F	2096/2098	1801-3136	1336bp	s	agatctACGGAGGAATCTTACTAACAGG	ccatggCCTTTCATTCTGAGGTAGTGTC
16	Synthetic	2010/2012	2150-1801	350bp	as	ccatggTGACCAACCGCAACCAGGAC	agatctAGGTCCAGGATGGTCTTGTTC
17	Synthetic	2010/2013	2648-1801	848bp	as	ccatggTGACCAACCGCAACCAGGAC	agatctGGATGCGGTCCTTGTTCAGG
18	Synthetic	2010/2099	2823-1801	1023bp	as	ccatggTGACCAACCGCAACCAGGAC	agatctTTGGTCTTGTCGTGGAACACC
19	Synthetic	2010/2100	3136-1801	1336bp	as	ccatggTGACCAACCGCAACCAGGAC	agatctAACAGAAGGTGGGGTCCTGC
20	Synthetic	2011/2013	2648-2125	524bp	as	ccatggAACAAGACCATCCTGGACCTG	agatctGGATGCGGTCCTTGTTCAGG
21	Synthetic	2011/2099	2823-2125	699bp	as	ccatggAACAAGACCATCCTGGACCTG	agatctTTGGTCTTGTCGTGGAACACC
22	Synthetic	2011/2100	3136-2125	1012bp	as	ccatggAACAAGACCATCCTGGACCTG	agatctAACAGAAGGTGGGGTCCTGC
23	G_F	1214/1215	14-1786	1773bp	s	agatctCCATCTTCAGCTCCAGACAG	ccatggCTGGCAATCTCTGGAGTTAG
24	G_F	2031/1797	2150-1801	350bp	as	ccatggCGTAAGAATCCTACTAACAGAAG	agatctGAGTTTTCTTTATTGTGTCTACTTCC
25	G_F	1218/1219	2648-2125	524bp	as	ccatggGGAAGTAGACACAATAAAGAAAACTC	agatctCTGGTGTAATTCTGATAGGCTTG
26	G_F	1218/1220	2823-2125	699bp	as	ccatggGGAAGTAGACACAATAAAGAAAACTC	agatctTTCGTGGAGAGATAATGCGTG
27	G_F	1218/1221	3136-2125	1012bp	as	ccatggGGAAGTAGACACAATAAAGAAAACTC	agatctCCTTTCATTCTGAGGTAGTGTC
28	F	2036/1797	2150-1801	350bp	as	ccatggGAGATTACAAGATGGTGAAAGG	agatctGAGTTTTCTTTATTGTGTCTACTTCC
29	F	2036/1219	2648-1801	848bp	as	agatctGAGTTTTCTTTATTGTGTCTACTTCC	agatctCTGGTGTAATTCTGATAGGCTTG
30	F	1218/1219	2648-2125	524bp	as	ccatggGGAAGTAGACACAATAAAGAAAACTC	agatctCTGGTGTAATTCTGATAGGCTTG
31	F	1218/1220	2823-2125	699bp	as	ccatggGGAAGTAGACACAATAAAGAAAACTC	agatctTTCGTGGAGAGATAATGCGTG

32	F	1218/1221	3136-2125	1012bp	as	ccatggGGAAGTAGACACAATAAAGAAAACTC	agatctCCTTTCATTCTGAGGTAGTGTC
33	A	2032/1797	2150-1801	350bp	as	ccatggCGTAAGAATCCTACTAACAGAAATC	agatctGAGTTTTCTTTATTGTGTCTACTTCC
34	A	1218/2138	2823-2125	699bp	as	ccatggGGAAGTAGACACAATAAAGAAAACTC	aacgcgtTTCGTGGAAAGATATTGTGTG
35	A	1218/2137	3136-2125	1012bp	as	ccatggGGAAGTAGACACAATAAAGAAAACTC	aacgcgtCCTTTCACTCTGAGGTAGTGTC
36	recoded	4320/4321	2823-2125	699bp	as	ccatggAGAGGTTGATACGATTAAAAAGACGC	agatctTTTGTTGAAAGGTAGTGTGTA

(B) Additional oligonucleotides used in this study.

DES837	TAATACGACTCACTATAGGG	3' sequencing primer for pTriplEX phage library (BD Biosciences)
DES886	AAGCGCGCCATTGTGTTGG	5' sequencing primer for pTriplEX phage library (BD Biosciences)
DES1141	GTAAAACGACGGCCAGTTTTTTTTTTTTTTT	M13(-20) and oligo-d(T), to prime cDNA from poly(A) mRNA
DES1165	CAATACAAGAACGGGAACAAC	L1 ORF2; paired with DES1166, nt 2858-2878, screen phage library
DES1166	ACCTTTGATGAGAATGAAGTGTC	L1 ORF2; paired with DES1165, nt 3269-3247 (AS)
DES1167	ACTAACAGGAACCAAGACCAC	L1 ORF1; paired with DES1168, nt 1814-1834, screen phage library
DES1168	GTTCATTTCCATCACCTGTTTGTATG	L1 ORF1; paired with DES1167, nt 2101-2076 (AS)
DES1249	TCGGAGGGCGAAGAATC	Hygromycin resistance gene, forward
DES1250	GTTGGCGACCTCGTATTG	Hygromycin resistance gene, reverse
DES1256	GACTTGTAACTCTTTAGCAGT	L1 ORF1, nt 2011-1991 (AS)
DES1847	AACAACACCCTGCTGAACG	smL1 ORF2, nt 3720-3738
DES1848	TGGGTGGTCAGGCTACTA	smL1 ORF2, nt 3922-3905 (AS)
DES1947	GGAAATGAACAAAACCATACTAGACC	L1 ORF1, nt 2092-2117
DES2784	CCTTCTTCTCAGCACCTC	L1 ORF2, nt 3538-3555
DES2790	GCTCTCTCCCGTTTTTTCTTG	L1 ORF2, nt 3898-3878 (AS)
DES2879	aagcggccgcTTCGTGGAGAGATAATGCGTG	NotI + L1 ORF1, nt 2823-2803 (AS)
DES2880	aagcggccgcGAGTTTTCTTTATTGTGTCTACTTCC	NotI + L1 ORF1, nt 2150-2125 (AS)
DES2881	aaggatccATTAGTCTGAACAGGTGAGAGG	BamHI + L1 ORF1, nt 1286-1307
DES2882	aaggatccTAAGAGAGCTTGCCAGCAGA	BamHI + L1 ORF1, nt 1636-1655
DES3062	TCGGTCGCCGCATACACTAT	*TEM1* reporter gene, forward
DES3063	GCAACTTTATCCGCCTCCATC	*TEM1* reporter gene, reverse
DES3353	AAACATACAAACAGGTGATGGAAATG	recoded L1 ORF1 (pJL3), nt 2073-2098
DES3354	AATTGTTGCTTCCTGTTATTTTAGTTG	recoded L1 ORF1 (pJL3), nt 2982-2956 (AS)
DES3818	gggatccTGGAACAGGCAGAAGCACAG	BamHI + smL1 ORF1, nt 1120-1140
DES3819	gggatccTGACCAACCGCAACCAGGAC	BamH1 + smL1 ORF1, nt 1812-1831
DES3820	ggctagcAGGTCCAGGATGGTCTTGTTC	NheI + smL1 ORF1, nt 2119-2098 (AS)
DES3821	ggctagcTTGGTCTTGTCGTGGAACACC	NheI + smL1 ORF1, nt 2800-2779 (AS)

Table S2. **Identification of AS L1 RIFTs.**

(A) **AS L1 fusion transcripts isolated from a mouse testis phage library.**

Chromosomal coordinates were based on the B6 reference genome, mm8 release. Transcripts that were initiated by polymorphic L1s, absent from the reference genome, were highlighted (*bold, italicized type*). Fusion transcript 2AS1-1 was expressed in the opposite orientation to AK155723. Some RIFTs already have been reported (1). L1 subfamilies were defined by RepeatMasker and have not been re-classified here, despite some known discrepancies in classifications (1). RIFTs bearing multiple names were identified independently, more than once.

fusion transcript name	GenBank acc. no.	chr.	L1 start coordinate	L1 stop coordinate	L1 subfamily	L1 orien.	fusion transcript span in genome, nt	overlapping cognate gene	GenBank acc. no.	ESTs over-lapping with L1	gene orien.	L1 location in gene	L1 AS fusion transcript coding	start of transcript in L1spa	stop of transcript in L1spa	orientation of transcript vs. native (overlapping) transcript	orientation of transcript vs. L1 template	transcript spliced	other comments
1ASII1	EF591873	8	EF591880		TF	+	42,649	N/A					ATG in AS L1	2241	1845	N/A; novel transcript	antisense	yes	polymorphic L1 template
2AS1-1	EU233991	4	73300011	73306261	TF	-	5,002		AK155723	CR517799	-	?	ATG in AS L1	2228	1855	AS to AK155723	antisense	yes	
4ASIII1-1	EU233992	7	6546454	6552141	F2	-	17,224	Usp29	NM_021323		+	intron6	Usp29	2190	1881	sense	antisense	yes	
4ASIII2-1 4AS1-1	EF591876	13	EF591881		TF	-	75,580	AK129128	NM_001081352		+	intron6	ATG in AS L1	2228	1859	sense	antisense	yes	
4ASIII4-2 Testisunspliced	EU234045	5	18957464	18961201	F/F2	+	2,845	ENSMUST00 000088516.2			+	intron	no; unspliced	1593	2023	sense	sense	no	not initiated in L1; terminated in L1
5AS1-1	EU233993	1	145473276	145479405	TF	-	30,463	Glrx2	NM_001038593		+	upstream	yes	2081	1839	sense	antisense	yes	
5ASII	EF591872	2	66106852	66112228	GF	+	30,052	Scn1a	NM_018733		-	intron1	ATG in AS L1	2223	1859	sense	antisense	yes	
5ASIII3-1	EU233994	13	105021323	105026446	F	+	81,840	Erbb2ip	NM_021563		-	intron1	yes	2221; 2036	2080; 1785	sense	antisense	yes	
6ASIII3-1 Testisunspliced	EU234041	12	94489362	94495492	TF	-	1,409				+		ATG in AS L1	2197	1433	N/A; novel transcript	antisense	no	
7ASII1	EU233995	13	8036636	8041441	GF	+	14,522		AK139843; AK141993				ATG in AS L1	2221	1859	AS to AK139843; AS to AK141993	antisense	yes	
7ASIII2-1 A	EF591875	12	78323703	78330141	TF	-	65,845	Fut8	NM_016893		+	intron6	ATG in AS L1	2187	1893	sense	antisense	yes	
7ASIII2-1 B	EF591874	12	78302248	78308516	TF	-	87,022	Fut8	NM_016893		+	intron5	yes	1998	1839	sense	antisense	yes	
7ASIII4-1 Testisunspliced	EU234044	14	123141801	123146971	A	+	2,270	Fgf14b	NM_207667		-	intron	unspliced	1593	2278	AS	sense	no	not initiated in L1; terminated in L1
7ASIII4-2	EF591871	2	43990003	43996650	TF	-	213,429	Arhgap15	NM_153820		+	intron9	ATG in AS L1	2224	1838	sense	antisense	yes	
8AS1-1	EF591877	13	EF591882		TF	+	59,553	Parp8	BC021881		-	intron6	yes	2211	1890	sense	antisense	yes	
9AS1-1	EF591879	16	95119882	95126090	TF	-	48,719						ATG in AS L1	2179	1893	N/A; novel transcript	antisense	yes	
11AS1-1	EU233997	16	488824642	48888091	GF	+	35,408	Dzip3	NM_027964		-	intron8	ATG in AS L1	2238	1892	sense	antisense	yes	
11ASII1 2ASII1	EF591878	15	EF591883		TF	-	56,583	Rnasen	NM_026799		+	intron11	ATG in AS L1	2225	1893	sense	antisense	yes	
10AS1-1	EU233996	2	83731030	83736749	F	+	16,622	Zswim2	NM_027964		-	intron3	ATG in AS L1	2240; 1984	2073; 1839	sense	antisense	yes	
12ASII	EU233998	1	107395525	107400953	GF	+	186,866	Pign	NM_013784		-	intron21	ATG in AS L1	2202	1839	sense	antisense	yes	see also CRL2196C02
70-8-1 10ASIII4-2	EU233999	3	69007284	69011494	F	+	26,484	Ift80	NM_026641	AK019542	-	intron13	ATG in AS L1	2206; 2036; 1999	2080; 2011; 1531	sense	antisense	yes	
70-7-1 Testisunspliced AK030137	EU234040	9	117351284	117357098	F/F2	-	3,629	Rbms3	NM_178660	AK030137	+		no; unspliced	2220; 2036; 1999	2080; 2011; 1540	AS to Rbms3 intron; sense to AK030137	antisense	no	
70-9-2 Testisunspliced	EU234043	7	104983758	104988672	GF	+	1,895				-		unspliced	1811	1569	N/A; novel transcript	antisense	no	
90-6-2 Testisunspliced	EU234042	12	94489362	94495492	TF	-	1,433	N/A					spliced within L1	2221; 1748	2085; 1433	N/A; novel transcript	antisense	yes	

Table S2. (B) **L1 fusion transcripts isolated from a mouse thymus cDNA phage library.**

Chromosomal coordinates were based on the B6 reference, mm8 release. L1 fusion transcripts identified from screens of a mouse thymus phage cDNA library were cloned and sequenced. L1 subfamilies were defined by RepeatMasker (www.repeatmasker.org) and have not been re-classified here (1). RIFTs bearing multiple names were identified independently, i.e. more than once. 61E and 21-4-1 are spliced AS L1 RIFTs. 21-3-2 and 21-1-2 are unspliced AS L1 RIFTs. Remaining transcripts were unspliced, sense-stranded cDNAs containing ORF1 sequences.

fusion transcript name	GenBank acc. no.	chr.	L1 start coordinate	L1 stop coordinate	L1 subfamily	L1 orien.	fusion transcript span in genome, nt	overlapping cognate gene	GenBank accession no.	ESTs overlapping with L1	gene orien.	L1 location in gene	fusion transcript coding	start of transcript in L1spa	stop of transcript in L1spa	orientation of transcript vs. native (overlapping) transcript	orientation of transcript vs. L1 template	other comments
61EThymus	EU234002	19	33626423	33631583	GF	+	6,307	AI747699	AK170332	partial overlap with AK135585	-	intron7	yes	2308	1531 (1479)	sense	antisense	unspliced in L1 and flanking genomic sequences; spliced in downstream exons
21-4-1Thymus	EU234003	10	57961513	57967477	F2	-	5,311	BC042726	BC042726		+	intron4	yes	2649	1587	sense	antisense	unspliced; sequences in intron of spliced gene
21-3-2 Thymusunspliced	EU234046	13	105021323	105026446	F	+	4,861	Erbb2ip	NM_021563	partial overlap with AK142040	-	intron1	no	3577	1531	sense	antisense	unspliced; sequences in introns of spliced gene
21-1-2 Thymusunspliced	EU234047	Y	456407	462013	F	+	4,820	Uty	BC053061		-	intron22	no	5106	1540	sense	antisense	unspliced; sequence entirely within intron of spliced gene; unusual promoter site within L1
61B Thymusunspliced	EU234048	15	42474518	42481162	TF	-	3,648	Angpt1	NM_009640		-	intron1	no	609; 910	697; 2059	sense	sense	unspliced; sequence in intron of spliced gene; promoter outside of L1; terminates in L1
(22-1-2) 26-2-1 Thymusunspliced	EU234049	18	5725792	5731219	F2/A	+	2,488	Zfhx1a	NM_011546		+	intron2	no	1593	2849	sense	sense	unspliced; sequence in intron of spliced gene; transcript entirely within L1
22-2-2 Thymusunspliced	EU234050	18	64036600	64041808	GF	+	1,666	WDR7 homolog	AK154451		+	intron21	yes	427; 1470	550; 2057	sense	sense	unspliced; sequence in intron of spliced gene promoter outside of L1; terminates in L1
26-5-2 Thymusunspliced	EU234051	2	37852416	37857621	F	+	5,633	Dennd1a	NM_146122	AK038066	-	intron8	no	1344	2300	antisense	sense	unspliced; sequence in intron of spliced gene; promoter outside of L1; terminates in L1; no poly(A) tail
59B Thymusunspliced	EU234052	9	55523029	55525579	GF	-	2,400	AK173175 AK048755	NM_001081 341		-	intron22	yes	1823; 2081	2015; 2856	sense	sense	unspliced; sequence in intron of spliced gene promoter outside of L1; terminates in L1
61C Thymusunspliced	EU234053	6	33706217	33711587	A	+	2,399	Exoc4	AK039983		+	intron11	yes	1540	2856	sense	sense	unspliced; sequence in intron of spliced gene; promoter outside of L1; terminates in L1
(63B) 65C Thymusunspliced	EU234054	1	88606392	88611310	GF	+	1,719	4930429A22 Rik	AK031180		+	intron5	yes	1540	2848	sense	sense	unspliced; sequence in intron of spliced gene; promoter outside of L1; terminates in L1

Table S2. (C) **AS L1 fusion transcripts from cell line CRL2196 and/or B6 and DBA/2J testes identified by RT-PCR.**

Primers for RT-PCR were designed based on “typical” AS L1 RIFTs observed initially in phage library screens. Total RNA from adult testis was used for RT-PCR. Candidate fusion transcripts were cloned and sequenced. Several fusion transcripts were identified from genomic regions lacking annotated genes or expressed sequence tags (EST). Two clones (DBA 2A12 and DBA B08) initiated from polymorphic L1s present in DBA/2J, absent from the reference genome. AS L1 RIFTs bearing multiple names were identified independently more than once.

fusion transcript name	GenBank accession no.	chr.	L1 start coordinate	L1 stop coordinate	L1 subfamily	L1 orien.	fusion transcript span in genome, nt	overlapping cognate gene	GenBank accession no.	gene orien.	L1 location in gene	fusion transcript coding	start of transcript in L1spa	stop of transcript in L1spa	orientation of transcript vs. native (overlapping) transcript	orientation of transcript vs. L1 template	fusion transcript splicing	other comments
CRL2196C02	EU234001	1	107395525	107400953	GF	+	47,812	Pign	AK132928	-	intron	yes	2011; 1530	1531; 1488	sense	antisense	yes (also in L1)	contains Lx in sense orientation (homologous to L1spa at 5819- 6679); ATG in Lx
CRL2196C10	EU234000	1	175854586	175859907	A	+	6,737	Ifi205	NM_172648	-	Intron 4	yes	1978	1593	sense	antisense	yes	Jonathan Keller
AK015524B	EU234039	1	140932925	140939189	TF	-	48,802	BC042698/ Dennd1b	NM_181347	+	intron10	ATG in AS L1	2221	1864	sense	antisense	yes	
B6 2B11	EU234011	7	83659505	83664656	GF	+	8,394	1700026 D08Rik	NM_029335	-	Intron 4	ATG in AS L1	2011	1835	sense	antisense	yes	
B6 2C04	EU234012	2	78748984	78755157	A	-	799	N/A		N/A	N/A	ATG in AS L1	2011; 1889; 1633	1968; 1838; 1538	N/A	antisense	yes (also in L1)	
B6 2C06	EU234013	1	24908585	24909777	F	+	1,768			N/A	N/A	yes	2011	1870	N/A	antisense	yes	
B6 2D07	EU234014	14	119878916	119885063	A	-	878					ATG in AS L1	2011; 1633	1840; 1538	N/A	antisense	yes (also in L1)	
B6 2E10	EU234015	18	44415203	44420652	GF	+	124,494	9530002K18	AK020535	-	5' intergenic region	ATG in AS L1	2009	1873	sense	antisense	yes	some ASL1 fusion transcript exons are novel; others shared with 9530002K18Rik
B6 B06	EU234016	17	89660065	89665291	L1VL1	+	50,630	N/A		N/A	N/A	ATG in AS L1	2011	1878	N/A	antisense	yes	
B6 B05	EU234017	15	61112866	61119031	F2	-	105,062	AK132805	AK132805		N/A	yes	2011; 1889	1968; 1836	sense	antisense	yes (also in L1)	overlap with AK132805
B6 B07<br>B6 C01<br>B6 2C01<br>DBA2D06	EU234018	9	20105039	20110504	GF	+	1,666	Zfp560	AK141494; AK009475	-	Intron 1	ATG in AS L1	2011	1839	sense	antisense	yes	
B6 C02	EU234019	4	112323142	112329580	L1_Mus1	-	17,878	Skint5	NM_001103199.1	-	2 splices in L1	yes	2057; 2000	2043; 1968	antisense	antisense	yes (also in L1)	exons overlap with Skint5 introns (AS)
B6 C03	EU234020	13	108552699	108554171	GF	+	6,482						2011	1841	N/A	antisense	yes	
B6 E02	EU234021	13	101218004	101223936	A	-	14,489	Smn1	BC045158	+	upstream	yes	2011	1835	sense	antisense	yes	
B6 E07	EU234022	16	77728359	77729431	F2	-	6,480						2011	1836	N/A	antisense		
DBA C07	EU234023	6	123097691	123104052	A	-	6,488	Clec4a2	NM_011999	+	Intron 3	ATG in AS L1	2011	1910	sense	antisense	yes	
DBA C02	EU234024	16	77728359	77729431	F2	-	6,442					ATG in AS L1	2011	1836	N/A	antisense	yes	
DBA C05	EU234037	13										ATG in AS L1	2011	1839	N/A	? (polymorphic L1?)	yes	possibly spliced transcript switching order of exons; or polymorphic L1 in DBA/2J
DBA B10	EU234025	18	70613427	70619844	A	-	7,152	Stard6	NM_029019	+	Intron 6 (2 splices in L1)		2011; 1889	1968; 1838	sense	antisense	yes (also in L1)	
DBA A06<br>DBA 2A04	EU234026	8	132024756	132031387	TF	-	34,271	1700008F21 Rik	NM_029292	+	Intron 5	ATG in AS L1	2011	1869	sense	antisense	yes	
DBA A11	EU234027	4	92557585	92563957	F3	-	17,878					ATG in AS L1	2011	1881	sense but minimally overlapping	antisense	yes	minimal overlap with exon from AK149399
DBA D11<br>DBA C12<br>DBA B09<br>DBA D10	EU234028	6	123097691	123104052	A	-	5,004	Clec4a2	BC006623; NM_011999	+	Intron 3	ATG in AS L1	2011	1881	sense	antisense	yes	
DBA A02	EU234029	3	102961728	102967399	GF	+	19,633	Sycp1	NM_011516	-	Intron 30	ATG in AS L1	2011	1892	sense	antisense	yes	
DBA 2E01	EU234030	11	54224849	54231970	TF	-	36,295	BC107369	BC107369	+	Intron 3	ATG in AS L1	2011	1892	sense	antisense	yes	
DBA 2C06	EU234031	13	78776733	78780209	A	-	3,746	AK133349	AK133349	+	2 splices in L1	spliced AK133349	2011	1869	sense	antisense	yes (also in L1)	overlap with AK133349 which is unspliced
DBA 2C05	EU234032	8	82655830	82662260	TF	-	15,586	Anapc10	NM_026904	+	Intron 4	ATG in AS L1	2011	1839	sense	antisense	yes	
DBA 2A03	EU234033	16	62038151	62040456	A/F2		7,469					ATG in AS L1	2011	1868	N/A	antisense	yes	
DBA 2A12	EU234034	9										ATG in AS L1	2011	1888	N/A	? (polymorphic L1?)	yes	
DBA 2B08	EU234035	10	17714696	17717225	F	+	14,850	(3110003A17 Rik)	NM_028440	-	5' intergenic region	yes	2136; 2035	2078; 1969	sense	antisense	yes	
DBA 2D08	EU234036	X	42871189	42873888	A	+	11,829					yes	N/A	N/A	N/A	antisense	yes (also in L1)	
DBA B08	EU234038	11				-		B3gnt1	NM_016888	-		ATG in AS L1	2011	1888	antisense (nonoverlapping)	? (polymorphic L1?)	yes	exons overlap with AS B3gnt1 introns
Ube3a fusion	EU234010	7	59162403	59168136	TF	-	3,064	Ube3a	NM_011668	+	intron11	yes	1957	1892	sense	antisense	yes	
Suclg2 fusion	EU234009	6	95623528	95629210	GF	+	181,456	Suclg2	NM_011507	-	intron1	ATG in AS L1	1957	1892	sense	antisense	yes	integrant (pseudogene) at chrX:152355677-152358910; good example of fusion proteins
Herc2 fusion	EU234008	7	55943138	55949786	TF	-	146,820	Herc2	NM_010418	+	intron4	ATG in AS L1	1957	1893	sense	antisense	yes	overlap of L1 exon and gene exon 3' sequences
Fgf17 fusion	EU234007	16	28106394	28111820	GF	+	29,517	Fgf12	NM_010199	-	intron4	ATG in AS L1	1957	1892	sense	antisense	yes	
Amph fusion	EU234004	13	19066760	19073230	TF	-	73,582	Amph	NM_175007	+	intron2	ATG in AS L1	1957	1886	sense	antisense	yes	
Antxr2 fusion	EU234005	5	98229028	98235131	GF	+	105,593	Antxr2	NM_133738	-	intron7	ATG in AS L1	1920	1845	sense	antisense	yes	
Car8 fusion	EU234006	4	8118895	8124119	GF	+	51	Car8	NM_007592	-	intron 3	ATG in AS L1	1957	1891	sense	antisense	yes	

Table S2. (D) **Computational identification of L1 fusion transcripts as full-length RIKEN ESTs.**

Transcripts in public mouse EST libraries were identified by BLASTN alignments; see text for details. Unannotated clones lacked information about overlapping cognate genes. All transcripts identified in this table were spliced. The highlighted clones (*gray*) are 17 clones originally identified by Zemojtel et al., 2007 (2).

RIKEN clones	Chr.	start (mm8)	stop (mm8)	L1 subfamily	L1spa start	L1spa stop	L1 orien.	fusion transcript span in genome, nt	overlapping cognate gene	Genbank_ accession	encoded protein	tissue	gene oren.	L1 location within gene
AK015008	10	111571425	111578851	L1Md_T	2242	1837	-	43,142	Caps2	NM_178278	BAC25455	testis	+	intron3
AK007235	8	96368470	96374755	L1Md_T	2205	1839	-	7,556	Ces7	NM_001003951	BAB24908	testis	+	upstream
AK015524	1	140932925	140939189	L1Md_T	2221	1893	-	48,802	BC042698	NM_181347	BAB29881	testis	+	intron10
AK015778	11	11526694	11531497	L1Md_F	2228	1839	+	38,602				testis		
AK131682	11	11526694	11531497	L1Md_F	2242	1839	+	4,117				testis		
AK015845	3	142962687	142968944	L1Md_F2	2202	1839	-	10,139				testis		
AK016072	16	75835067	75840241	L1Md_Gf	2228	1838	+	94,076	Samsn1	NM_023380	P57725	testis	-	upstream
AK132741	12	90764000	90770216	L1Md_F2	2188	1837	-	3,427				testis		
AK132928	1	107395525	107400953	L1Md_Gf	2228	1839	+	48,020	Pign	NM_013784	Q9R1S3	testis	-	intron21
AK161293	6	57557930	57564107	L1Md_T	2206	1838	-	29,470				testis		
AK135585	19	33626423	33631583	L1Md_Gf	2221	1839	+	5,960				testis		
CA463860	12	34351760	34357164	L1Md_Gf	2215	1838	+	1,700				testis		
BB614971	18	7465319	7466485	L1Md_F	2228	1937	+	17,689	Mpp7	NM_001081287	NP_001074756	testis	-	intron4
BB615298	8	46081938	46088182	L1Md_T	2228	1839	-	3,012				testis		
CF106266	14	80130384	80134274	L1Md_A	2132	1839	+	87,235				testis		
AK015266	10	44426542	44431581	L1Md_Gf	2194	1570	+	8,132				testis		
AK015267	5	45249224	45255386	L1Md_F2	2226	1838	-	12,508				testis		
AK015548	12	66100620	66101658	L1Md_F	2221	1841	-	4,008				testis		
AK076999	4	30595576	30601899	L1Md_F2	2226	1540	-	91,777				testis		
AK015559	1	24908585	24909777	L1Md_F	2235	224	+	1,159				testis		
AK077067	13	78776733	78780209	L1Md_A	2233	1540	-	3,943				testis		
AK006905	18	70635500	70640669	L1Md_Gf	2221	1839	+	2,366	Poli	NM_011972	Q6R3M4	testis	-	intron9
AK076828	13	76351495	76353381	L1VL1	2163	2116	+	13,655	Rhobtb3	NM_028493	Q9CTN4	testis	-	intron8

Table S2. (E) **Additional mouse ESTs containing AS L1 sequences in 5' ends**. A subset of EST clones included spliced exons but no splicing within L1 sequences (*highlighted in yellow*). These transcripts were identified from public mouse EST libraries by BLASTN alignments; see text for details. Unannotated clones lacked information about overlapping cognate genes.

RIKEN fusion transcript, GenBank acc. no.	chr.	Description	clone ID no.	sequenced	subfamily	L1 length, nt
CJ067405	13	RIKEN full-length enriched mouse cDNA library, C57BL/6J 8 cells embryo 8 cells Mus musculus cDNA clone E860002I03 5-	E860002I03	5, 3 end	L1VL1	4254
BB622436	16	RIKEN full-length enriched, adult male olfactory brain Mus musculus cDNA clone 6430404D24 5-	6430404D24	AK032159	L1VL1	5263
BB655922	14	RIKEN full-length enriched, 12 days embryo spinal ganglion Mus musculus cDNA clone D130046G19 5-	D130046G19	AK051402	L1VL1	1715
BB616604	12	RIKEN full-length enriched, adult male testis Mus musculus cDNA clone 4932422P09 5-	4932422P09	5, 3 end	L1VL1	2999
BB609902	5	RIKEN full-length enriched mouse cDNA library, C57BL/6J lung male adult Mus musculus cDNA clone 1200008I23 5-	1200008I23	5, 3 end	L1VL2	1066
CJ162777	9	RIKEN full-length enriched mouse cDNA library, Rathke-s pouches 14.5 days embryo Mus musculus cDNA clone M130004H22 5-	M130004H22	5, 3 end	F2	6293
AA789378	13	vv93e11.r1 Stratagene mouse skin (#937313) Mus musculus cDNA clone IMAGE:1229996 5-	IMAGE 1229996	5, 3 end	F2	6210
CJ046686	6	RIKEN full-length enriched mouse cDNA library, C57BL/6J testis male adult Mus musculus cDNA clone 4921526E09 5-	4921526 E09	AK132683	F2	5934
CJ058240	10	RIKEN full-length enriched mouse cDNA library, C57BL/6J thymus 0 day neonate Mus musculus cDNA clone A430102J02 5-	A430102J02	5, 3 end	F2	6224
CJ046787	9	RIKEN full-length enriched mouse cDNA library, C57BL/6J testis male adult Mus musculus cDNA clone 4922504A12 5-	4922504A12	AK132735	F2	6293
BB616227	4	RIKEN full-length enriched, adult male testis Mus musculus cDNA clone 4931422A14 5-	4931422A14	AK076999	F2	6324
BB616730	2	RIKEN full-length enriched, adult male testis Mus musculus cDNA clone 4932434A08 5-	4932434A08	5, 3 end	F2	6159
BB616883	9	RIKEN full-length enriched mouse cDNA library, C57BL/6J testis male adult Mus musculus cDNA clone 4932702M09 5-	4932702M09	AK030137	F2	4714
BY715427	2	RIKEN full-length enriched, adult male testis Mus musculus cDNA clone 4930522P10 5-	4930522P10	AK015873	F3	5617
CJ046771	13	RIKEN full-length enriched mouse cDNA library, C57BL/6J testis male adult Mus musculus cDNA clone 4922503G03 5-	4922503G03	AK132727	F3	5964
BB641970	10	RIKEN full-length enriched, 10 days neonate cortex Mus musculus cDNA clone A830084P16 5-	A830084P16	AK044056	F3	6200
BB637513	13	RIKEN full-length enriched, adult male aorta and vein Mus musculus cDNA clone A530081E06 5-	A530081E06	AK041083	F	820
BY716756	19	RIKEN full-length enriched, adult male testis Mus musculus cDNA clone 4933436E20 5-	4933436 E20	AK017078	F	703
BB615444	5	RIKEN full-length enriched, adult male testis Mus musculus cDNA clone 4930509B07 5-	4930509B07	AK132908	F	1255
BB645345	7	RIKEN full-length enriched mouse cDNA library, C57BL/6J adipose male 4 days neonate Mus musculus cDNA clone B430110I16 5-	B430110I16	AK046587	F	2472
BY714480	3	RIKEN full-length enriched, adult male testis Mus musculus cDNA clone 4921524P20 5-	4921524P20	AK019542	F	4211
BB641176	15	RIKEN full-length enriched, 10 days neonate cortex Mus musculus cDNA clone A830009L17 5-	A830009L17	AK043579	F	4127
BY729029	1	RIKEN full-length enriched, 7 days embryo whole body Mus musculus cDNA clone C430022K04 5-	C430022K04	5, 3 end	F	3718
BB614554	2	RIKEN full-length enriched mouse cDNA library, C57BL/6J testis male adult Mus musculus cDNA clone 4921510J19 5-	4921510J19	5, 3 end	F	1619
BY714877	13	RIKEN full-length enriched, adult male testis Mus musculus cDNA clone 4930435F18 5-	4930435F18	AK015323	F	1964
BB614452	11	RIKEN full-length enriched mouse cDNA library, C57BL/6J testis male adult Mus musculus cDNA clone 4932416O13 5-	4932416o13	AK133208	F	3863
AW824969	13	us09f03.y1 Soares_NMGBC_B-cell Mus musculus cDNA clone IMAGE:3166589 5-	IMAGE3166589	5, 3 end	Mus1	6223
BB615657	15	RIKEN full-length enriched mouse cDNA library, C57BL/6J testis male adult Mus musculus cDNA clone 4930542I17 5-	4930542I17	AK132982	Mus1	6120
BB641591	4	RIKEN full-length enriched, 10 days neonate cortex Mus musculus cDNA clone A830034C17 5-	A830034C17	AK043797	LX4B	3432
BB616626	9	RIKEN full-length enriched mouse cDNA library, C57BL/6J testis male adult Mus musculus cDNA clone 4932427D17 5-	4932427D17	5, 3 end	A	4708
BB646099	13	RIKEN full-length enriched, 10 days neonate cerebellum Mus musculus cDNA clone B930009D02 5-	B930009D02	AK080998	A	5034
BY306964	9	RIKEN full-length enriched, 12.5 days embryo Rathke-s pouches Mus musculus cDNA clone K920012K13 5-	K920012K13	5, 3 end	A	2769
BY307063	9	RIKEN full-length enriched, 12.5 days embryo Rathke-s pouches Mus musculus cDNA clone K920013C13 5-	K920013C13	5, 3 end	A	2769
CJ055932	2	RIKEN full-length enriched mouse cDNA library, C57BL/6J cerebellum 16 days neonate Mus musculus cDNA clone 9630026B05 5-	9630026B05	5, 3 end	A	6393
BB641162	8	RIKEN full-length enriched, 10 days neonate cortex Mus musculus cDNA clone A830008M02 5-	A830008M02	5, 3 end	A	6606
BY715083	17	RIKEN full-length enriched, adult male testis Mus musculus cDNA clone 4930470H14 5-	4930470H14	AK015535	A	5164
BY716637	2	RIKEN full-length enriched, adult male testis Mus musculus cDNA clone 4933427J07 5-	4933427J07	AK016956	A	3459
BB648069	X	RIKEN full-length enriched, 16 days embryo head Mus musculus cDNA clone C130053F07 5-	C130053F07	AK048370	A	6392
BY715636	1	RIKEN full-length enriched, adult male testis Mus musculus cDNA clone 4930545H10 5-	4930545H10	AK016047	A	1025
BY716764	1	RIKEN full-length enriched, adult male testis Mus musculus cDNA clone 4933436I20 5-	4933436I20	AK016047	A	1025
BB573692	13	RIKEN full-length enriched, adult male testis (DH10B) Mus musculus cDNA clone 4933400A22 5-	4933400A22	AK077067	A	3477
BB616797	13	RIKEN full-length enriched, adult male testis Mus musculus cDNA clone 4932439N19 5-	4932439N19	AK133349	A	3477
BB617027	18	RIKEN full-length enriched mouse cDNA library, C57BL/6J testis male adult Mus musculus cDNA clone 4933412A06 5-	4933412A06	AK030177	A	1282
BY715415	14	RIKEN full-length enriched, adult male testis Mus musculus cDNA clone 4930521O11 5-	4930521o11	AK015864	A	2492
BY714825	10	RIKEN full-length enriched, adult male testis Mus musculus cDNA clone 4930431F10 5-	4930431F10	AK015266	GF	5092
CJ159974	4	RIKEN full-length enriched mouse cDNA library, Rathke-s pouches 14.5 days embryo Mus musculus cDNA clone K720041H22 5-	K720041H22	5, 3 end	GF	1611
CJ160006	4	RIKEN full-length enriched mouse cDNA library, Rathke-s pouches 14.5 days embryo Mus musculus cDNA clone K720045M06 5-	K720045M06	5, 3 end	GF	1611
CJ160063	4	RIKEN full-length enriched mouse cDNA library, Rathke-s pouches 14.5 days embryo Mus musculus cDNA clone K720049P11 5-	K720049P11	5, 3 end	GF	1611
BB616468	5	RIKEN full-length enriched mouse cDNA library, C57BL/6J testis male adult Mus musculus cDNA clone 4932416G22 5-	4932416G22	AK030034	GF	1316
BB616213	1	RIKEN full-length enriched mouse cDNA library, C57BL/6J testis male adult Mus musculus cDNA clone 4931420K07 5-	4931420K07	AK029879	GF	5468
BB614575	11	RIKEN full-length enriched, adult male testis Mus musculus cDNA clone 4921513H21 5-	4921513H21	5, 3 end	GF	5707
BB629795	15	RIKEN full-length enriched mouse cDNA library, C57BL/6J vagina female adult Mus musculus cDNA clone 9930010I05 5-	9930010I05	AK036791	TF	6431
CN690765	2	NIA Mouse E10.5 whole embryo cDNA library (Long) Mus musculus cDNA clone NIA:E0300E06 IMAGE:30858965 5-	IMAGE30858965	5 end	TF	4701
BB614622	3	RIKEN full-length enriched, adult male testis Mus musculus cDNA clone 4921521K05 5-	4921521K05	5, 3 end	TF	6093
BB616381	1	RIKEN full-length enriched mouse cDNA library, C57BL/6J testis male adult Mus musculus cDNA clone 4932411L15 5-	4932411L15	AK029971	TF	5683
CJ168023	6	RIKEN full-length enriched mouse cDNA library, Rathke-s pouches 14.5 days embryo Mus musculus cDNA clone M130022C12 5-	M130022C12	5, 3 end	TF	6382
BB614889	17	RIKEN full-length enriched mouse cDNA library, C57BL/6J testis male adult Mus musculus cDNA clone 4930404G17 5-	4930404G17	5, 3 end	TF	6863

Table S3. **AS L1 RIFTs were expressed genome-wide, as identified by a novel assay using exon microarray.**

Presented here is a list of genes expressed as spliced AS L1-gene RIFTs in adult B6 testis. For inclusion on this list, we required annotated RefSeq genes to be represented on an Affymetrix mouse exon microarray, at least five consecutive exons must show high signal intensities upon hybridization with AS L1 RIFT RT-PCR products, a reference full-length AS L1 element must reside within 100 kb of the high intensity probe sites mapped to the genome, and the initiating L1 must reside within 30 kb of the RefSeq gene. These criteria were chosen to reduce false positive calls of AS L1 RIFTs. Highlighted (*yellow*) are gene templates for AS L1 RIFTs that were also identified by another method (1).

Aox3l1	*Prkdc*	*Tek*
Spag16	*Zbtb20*	*Car8*
Glrx2	*Slc9a10*	4930579C15Rik
Astn1	*Morc1*	*Grk4*
Kifap3	2310008H04Rik	4933428G09Rik
Fmn2	*Lamp3*	*Rufy3*
Tram2	*Atp13a4*	*Pitpnb*
Smap1	2310005G13Rik	A330021E22Rik
Bai3	*Prss7*	*Adam22*
Hecw2	*Park2*	*Gm440*
Pign	*Supt3h*	*Cd36*
Ptpn4	*Emr1*	*Stk32b*
Il20	AW061290	*Lcorl*
Epm2a	*Crisp2*	*Tmprss11d*
Slc35f1	*Tbc1d5*	*Tmprss11b*
Tpd52l1	*Pcdha4*	*Cnot6l*
Lace1	*Adamts19*	*Antxr2*
Tmem16d	*Pias2*	*Glmn*
BC067068	*Ccny*	*Rfc3*
Trhde	4921528I01Rik	*Ccdc132*
Slc16a7	*Epb4.1l4a*	*Phf14*
Fancl	*Stam*	*Grid2*
Havcr1	*Slc39a12*	*Smarcad1*
Tanc2	*Arhgap15*	*Nr2c2*
Dgkb	*Ccdc34*	*Asns*
Pnpla8	*Itga8*	*Asz1*
Fut8	*Nsun6*	*Iqub*
Gphn	*Dennd1a*	*Tpk1*
4921509E07Rik	*Scn1a*	*Pde1c*
Lyst	*Zswim2*	*Ctnna2*
Mctp1	*Elp4*	*Otud7a*
Smn1	*Hao1*	*Prcp*
5033411D12Rik	*Tasp1*	*Nars2*
4930486L24Rik	OTTMUSG00000015730	*Abca14*
Rhobtb3	*Dnajc5b*	LOC100047837
Erbb2ip	*Spata16*	*Ntrk3*
Wapal	*Pglyrp3*	*Tpte*
Pcca	4921515J06Rik	*Tusc3*
4930452B06Rik	*Dpyd*	*Csmd1*
1700129C05Rik	*Unc5c*	*Ccdc7*
Fndc3a	*Etfdh*	*Naalad2*
Diap3	*Sycp1*	*Dock3*
Klhl1	*Gstcd*	*Maoa*
Colec10	*Pkn2*	*Gria3*
Khdrbs3	4930473A06Rik	*Asb9*
Rspo2		

Table S4. **Alignment of splice donor and splice acceptor sites in fusion transcripts.**

Genomic template sequences flanking the first introns of antisense L1 fusion transcripts, including splice donor and splice acceptors, were aligned. The spliced intronic sequences are highlighted (*red*) and canonical splicing donor (GT) and acceptor (AT) sites are underlined. Exon sequences are shown in capital letters (*black*). Splice donor sites were grouped into arbitrary consensus types I – VIII based on intron alignments, and splice position is indicated, based on L1spa coordinates (*splice type, position*).

ASL1 fusion transcripts	splice donor		splice acceptor	splice type, position
1ASII1	TCTGGTGATGgtgagtggt	....	tctccccagCCTCTCACCC	I 1838
2AS1-1	TCTAGTGATGgtgagtggt	....	ctattccagAAAGTAGATC	I
4ASIII2-1/4AS1-1	TTTGGTGATGgtgagtggt	....	tttcaccagGTGGCTCGGA	I
4ASIII1-1	TCTGATGATGgtgagtggt	....	atcccctagGGATAGGAAT	I
5AS1-1	TCTGGTGATGgtgagtggt	....	ctgctacagACAATACAGC	I
5ASII	TCTGATGATGgtgagtggt	....	tatgcccagAAACTCAACG	I
10AS1-1	TCTGTTGATGgtgagtggt	....	tttcaccagCTGCTTTTCA	I
7ASII1	TCTGATGATGgtgagtggt	....	ggttttcagGATtAGAATA	I
7ASIII2-1B	TCTGGTGATGgtgagtggt	....	ctttcacagGTCTATCATG	I
7ASIII4-2	TCTGGTGATGgtgagtggt	....	tgtttttagGCCTAGACGT	I
12ASII	TCTGATGATGgtgagtggt	....	tgttatcagAACCTTCACT	I
Pign	TCTGATGATGgtgagtggt	....	ttcttgcagCCTCTTCTCT	I
11ASII1/2ASII1	AGGTTCTCAGgtgtgttgg	....	tcacccaagATGGAGGCAA	II 1892
8AS1-1	AGGTTCTCAGgtgtgttgg	....	ttctggcagGTGAACTATG	II
9AS1-1	AGGTTCTCAGgtgtgttgg	....	ttttcttagAGAGAAACAA	II
7ASIII2-1A	AGGTTCTCAGgtgtgttgg	....	cactggcagAATCCTAAGG	II
11AS1-1	AGGTTCTCAGgtgtgttgg	....	aaattctagGATAGTCTCA	II
70-8-1/10ASIII4-2	ATTAAACTTGgtataataa	....	atttttaagGAACCATGGT	III 1540
5ASIII3-1	TTTGCCATCTgataatctc	....	agaccccctaTCCTCTGGCT	IV 1785
DBA 2A03	TCTGATGATGgtgagtggt	....	tttcttcagATTCTACTGA	I
B6 2B11	TCTGATGATGgtgagtggt	....	tgtttccagCTGTATCTAA	I
B6 2C04	TCTGAGGATGgtgagtggt	....	ctctttcagGGAAGGTGCA	I
B6 2C06	TCTGATGATGgtgagtggt	....	tttccacagAAACAAACTA	I
B6 2D07	TCTGATGATGgtgagtggt	....	ctctttcagGGAAGGTGCA	I
B6 B05	TCTGATGATGgtgagtggt	....	tatgtccagAGATTACATC	I
B6 B07/C01/2C01	TCTGATGATGgtgagtggt	....	gattaatagATAATATTTG	I
B6 C02	TTTGATGATGgtgagtggt	....	tcaagacagGGTTTCTCTG	I
B6 C03	TCTGATGATGgtgagtggt	....	tctctgcagCT*T*GTACAGT	I
B6 E07	TCTGATGATGgtgagtggt	....	tccctccagATGTCTACCA	I
DBA 2C06	TCTGATGATGgtgagtggt	....	tctggccagGAAAGTGCCC	I
DBA C02	TCTGATGATGgtgagtggt	....	tccctccagATGTCTACCA	I
DBA B10	TCTGATGATGgtgagtggt	....	ttgttgcagGACACACTCA	I
DBA 2C05	TCTGGTGATGgtgagtggt	....	tcttatcagCAACTTGAAT	I
DBA A11	TCTGATGATGgtgagtggt	....	acttcatagGTCAACAGAA	I
DBA D11/C12/B09/D10	TCTGATGATGgtgagtggt	....	tcctaacagCACCCCCCAT	I
DBA A02	AGGTTCTCAGgtgtgttgg	....	tttacatagGAATATACAG	II
DBA 2E01	AGGTTCTCAGgtgtgttgg	....	gctttacagGACCAGCCGA	II
B6 E02	GATGTGGTGAgtggtcttg	....	tcttcagagTGATGATTCT	V 1834
B6 2E10	GGAGTGCTGGattctgatg	....	atagtctacTTCCTATGGA	VI 1850
DBA C07	CAAGTGCTACgttctgatg	....	cccccaaagACAAAGTCTG	VI 1850
DBA A06/2A04	CCTGGACTGGgcgaagtgg	....	tgtctttagAAATATAAAC	VII 1869
DBA 2A12 (di)	GTTCTCAGGTgtgttgggg	....	gtacttcagATTATTTAAA	VIII 1890
DBA B08 (di)	GTTCTCAGGTgtgttgggg	....	attttgcagCTAAAATATC	VIII 1890
AK015008	TCTGGTGATGgtgagtggt	....	atttttcagGTCTTGTTTA	I
AK007235	TCTGGTGATGgtgagtggt	....	ttttttcagATTCTGGTGG	I

AK015778	TCTGATGATGgtgagtggt	………..	ttctttcagACCAGCGTAT	I
AK131682	TCTGATGATGgtgagtggt	………..	ttctttcagACCAGCGTAT	I
AK015845	TCTGATGATGgtgagtggt	………..	ctgctccagATTTCAAAGC	I
AK016072	TCTGATGATGgtgagtggt	………..	ttgtgctagGCCATAAAAT	I
AK0132741	TCTGATGATGgtgagtggt	………..	tctacccagGTATAGAATT	I
AK132928	TCTGATGATGgtgagtggt	………..	ttcttgcagCCTCTTCTCT	I
AK161293	TCTGGTGATGgtgagtggt	………..	tttttttagGACTGTACAG	I
AK135585	TCTGATGATGgtgagtggt	………..	caatttcagCTCCTCTTTG	I
AK015524	AGGTTCTCAGgtgtgttgg	………..	tatttcgagCTTCCCTAAA	II
AK015524B	AGGTTCTCAGgtgtgttgg	………..	aaatttcagTTTATTGATG	II
CF106266	TCTGATGATGgtgagtggt	………..	gttctttagACCAATCAAG	I
BB615298	TCTGGTGATGgtgagtggt	………..	gttgcacagATATATACAA	I
BB614971	TCTGATGATGgtgagtggt	………..	ttcttccagATTCATGAAA	I
CA463860	TCTGATGATGgtgagtggt	………..	ttcttgaagGAATGTACAA	I
Amph fusion	TCTGGTGATGgtgagtggt	………..	gtttttcagGCAGAGGGTA	I
Antxr2 fusion	TCTGATGATGgtgagtggt	………..	ccttttcagATATTAGCTC	I
Ube3a fusion	AGGTTCTCAGgtgtgttgg	………..	tcacattagGGAGTTCTGG	II
Sulg2 fusion	AGGTTCTCAGgtgtgttgg	………..	tctccctagGCCGGTCACT	II
Herc2 fusion	AGGTTCTCAGgtgtgttgg	………..	ttgtttcagATGTGAATGA	II
Fgf17 fusion	AGGTTCTCAGgtgtgttgg	………..	tttcttcagGATGTTTTTA	II

SUPPLEMENTARY FIGURE LEGENDS.

Fig. S1. **Additional reporter assays identify AS promoter activity within ORF1 of certain other L1 elements.**

Additional mouse L1 fragments (sources and coordinates, Table S1) were engineered upstream of the beta-lactamase *TEM1* reporter in the orientation indicated by arrows, and assayed for promoter activity (Fig. 1). Linearized DNAs containing the promoter-reporter cassette were transfected into cultured HeLa (human cervical carcinoma) and CRL-2196 (mouse spermatocyte) cells. Reporter expression was detected by staining cells with CCF2-AM, whose green fluorescent emission spectrum shifts to blue upon cleavage by beta-lactamase (3). Promoter activity scores (*colors in key, top*) were assigned to each strand-specific candidate tested. Fragments are mapped to L1spa coordinates (*arrows*). The sources of fragments and the cell lines used for transfection also are indicated.

Fig. S2. **Alignments of L1 subfamily and recoded ORF1 sequences spanning the AS promoter.**

(A) AS L1 subfamily consensus sequences, corresponding to the active AS promoter region centered on ORF1 (Fig. 1, Fig. S1), were generated for four subfamilies, T_F, G_F, A and FII by querying the mouse reference genome (UCSC mm10) using seed sequences L1G_F62, L1spa and L1Md_A2. In each case, >80 genomic elements were found that aligned at >97% identity to the query sequence. Consensus sequences were defined by majority rule. They were aligned to corresponding regions of smL1 (also called ORFeus) (4), and to our novel, recoded L1 in pJL3. Coordinates and the reference sequence were based on L1spa (L1 T_F). Predicted transcription factor binding sites were identified using TFSEARCH: Searching Transcription Factor Binding Sites (v. 1.3) (http://www.cbrc.jp/research/db/TFSEARCH.html). As shown here, they are underlined and labeled above the sequences. *Yellow highlights*: single nucleotide differences between subfamilies; *gray highlights*: differences between consensus elements and individual surrogates; *asterisks below aligned nucleotides*: conserved among all 6 aligned elements. (B) Plot of the fractional mismatch in 60 nt windows spanning the ORF1 region, presented in the sense orientation (based on L1spa reference sequence). *Arrow:* approximate location of AS promoter.

Fig. S3. **Genomic templates of AS L1 RIFTs.** (A) Schematic of an AS L1 integrant, located within an intron of an arbitrary gene. 5' UTR, 5' untranslated region; ORF1, open reading frame-1; LPR, length polymorphism region. (B) Schematics of diverse genomic templates and exons displaying the templates for specific AS L1 RIFTs, following a previously published format (5). Fusion AS L1-gene cDNAs were aligned to the B6 reference genome (UCSC browser, release mm8). Chromosomal sequences are represented by single horizontal lines, and spliced exons included in the cognate gene and/or RIFT are indicated by rectangles: (*black*) conventional transcript exons omitted from RIFTs; (*red*) first alternative first L1 exon; (*blue*) conventional transcript exons included in RIFTs; and (*green*) RIFT exons omitted from conventional transcripts. Also indicated are the cDNA clone and gene names (*left*) and the genomic length (bp) spanned by the L1 RIFT (*right*).

Fig. S4. **Tissue-specific expression of L1 RIFTs.**
RT-PCR was performed for 17 pairs of L1 fusion transcripts (*upper panel of each pair*) and their overlapping cognate genes (where applicable; *lower panels*). A mouse multiple tissue cDNA panel, generated from a mouse strain known to include indicated L1 variants, was used in this screen. The tissues are: 1, heart; 2, brain; 3, spleen; 4, lung; 5, liver; 6, skeletal muscle; 7, kidney; 8, testis; 9, 7-day embryo; 10, 11-day embryo; 11, 15-day embryo; 12, 17-day embryo; 13, H_2O. As a positive control, *GAPDH* transcripts were amplified by RT-PCR.

Fig. S5. **Logo bitmap of splice donor and acceptor sites**. Spliced fusion transcripts were aligned to genomic templates, and 10 nucleotides on either side of (A) splice donor and (B) splice acceptor sites were aligned. (See Table S4.) Logo bitmaps indicate that the consensus L1 fusion transcript splice sites follow well-characterized splicing patterns.

Fig. S6. **AS L1 promoter activity may contribute to reduced native L1 transcription.** To evaluate the effects of native AS ORF1 transcription upon transfected L1 retrotransposon transcript levels expressed from donor plasmids, we transiently transfected native, hybrid and fully synthetic elements. Transcript levels of the various L1 elements were measured by qRT-PCR assays of ORF2 levels, normalized to *Hygro*^{R} transcript levels (*y-axis*, presented as log2 of transcription concentration, based on delta Ct = –(Ct (ORF2) – Ct (Hygro))). *Categories*, *x-axis*: T_F, native L1spa from pTN201; recoded/T_F, neutral changes in A/T content of recoded ORF1 in pJL3; smL1/T_F, low A/T content of ORF1 swapped from smL1 into L1spa, resulting in a hybrid L1

donor, pMK28; smL1, fully synthetic L1 ORF1 and ORF2 in pCEP4/smL1. *Values*, mean of duplicates; *error bars*, range.

Fig. S7. **AS L1 transcripts limit retrotransposition by synthetic and native L1 elements *in trans*.** We studied the effects of AS RIFTs (expressed *in trans*) on (A) smL1 or (B) native L1 retrotransposition frequencies. (A) To generate AS smL1 RIFTs, first we directionally cloned four AS fragments derived from smL1 (*schematic at top*), i.e. mapping to corresponding L1spa coordinates 2119-1120 (PCR amplicon DES3820 x DES3818, Table S1); 2800-1120 (DES3821xDES3818); 2119-1812 (DES3820 x DES3819); and 2800-1812 (DES3821 x DES3819). These fragments were cloned into pCEP4 downstream of its strong CMV promoter. Each cloned construct was co-transfected into HeLa cells with the smL1 retrotransposition donor plasmid, pCEP4/smL1/Neo-AI. As positive and negative controls, the smL1 donor alone and empty pCEP4 alone were transfected into HeLa cells, respectively. After transfections, cells were plated at dilutions (i.e. 1x, 1/10, 1/100), selected on G418 for 2 weeks in a transient retrotransposition assay (6), and Neo^R colonies were stained and counted (Fig. 6). We observed non-linear saturation of colony numbers at the 1x plating density (not shown), so only the 1/10 and 1/100 dilutions are shown. (B) (*Schematic at top*) Three different AS native $L1T_F$ fragments (derived from L1spa template in pTN201) i.e. mapping to L1spa coordinates 2823-1286, 2150-1286 and 2150-1636, were amplified from L1spa using primers DES2879-DES2882; Table S1B). These fragments were directionally cloned into pCEP4 downstream of its strong CMV promoter. Each cloned construct was co-transfected into HeLa cells with the native L1spa (L1 T_F) donor plasmid, pTN201. As positive and negative controls, L1spa and empty vector pCEP4 were transfected alone, respectively. After transfection, equal numbers of HeLa cells were plated, selected on G418 for 2 weeks, and Neo^R colonies were stained and counted.

Fig. S8. **Minimal role of Dicer in regulating L1 retrotransposition.** Retrotransposition of various L1 elements was assayed in the presence or absence of Dicer. We quantified spliced *TEM1*, expressed from retrotransposed genomic integrants, using qRT-PCR of spliced *TEM1* transcripts. *Y-axis*: delta Ct = Ct (*TEM1*) – Ct (*GAPDH*). Higher Ct values correspond to lower transcript template concentrations. *X-axis categories*: T_F, native L1spa from pTN201; recoded/T_F, neutral changes in A/T content of recoded ORF1 in pJL3; smL1/T_F, low A/T content in ORF1 swapped from smL1 into L1spa (L1 T_F), resulting in a hybrid L1 donor, pMK28; smL1, fully synthetic L1 ORF1 and ORF2 in pCEP4/smL1. *Values*, mean of duplicates; *error bars*, range; *black bars*, HCT116 wildtype cells; *white bars,* HCT116 Dicer ex 5 -/- cells.

SUPPLEMENTARY REFERENCES

1. Akagi, K., Li, J., Stephens, R.M., Volfovsky, N. and Symer, D.E. (2008) Extensive variation between inbred mouse strains due to endogenous L1 retrotransposition. *Genome Res*, 18, 869-880.
2. Zemojtel, T., Penzkofer, T., Schultz, J., Dandekar, T., Badge, R. and Vingron, M. (2007) Exonization of active mouse L1s: a driver of transcriptome evolution? *BMC Genomics*, 8, 392.
3. Zlokarnik, G., Negulescu, P.A., Knapp, T.E., Mere, L., Burres, N., Feng, L., Whitney, M., Roemer, K. and Tsien, R.Y. (1998) Quantitation of transcription and clonal selection of single living cells with beta-lactamase as reporter. *Science*, 279, 84-88.
4. An, W., Han, J.S., Wheelan, S.J., Davis, E.S., Coombes, C.E., Ye, P., Triplett, C. and Boeke, J.D. (2006) Active retrotransposition by a synthetic L1 element in mice. *Proc Natl Acad Sci U S A*, 103, 18662-18667.
5. Peaston, A.E., Evsikov, A.V., Graber, J.H., de Vries, W.N., Holbrook, A.E., Solter, D. and Knowles, B.B. (2004) Retrotransposons regulate host genes in mouse oocytes and preimplantation embryos. *Dev Cell*, 7, 597-606.
6. Wei, W., Morrish, T.A., Alisch, R.S. and Moran, J.V. (2000) A transient assay reveals that cultured human cells can accommodate multiple LINE-1 retrotransposition events. *Anal Biochem*, 284, 435-438.

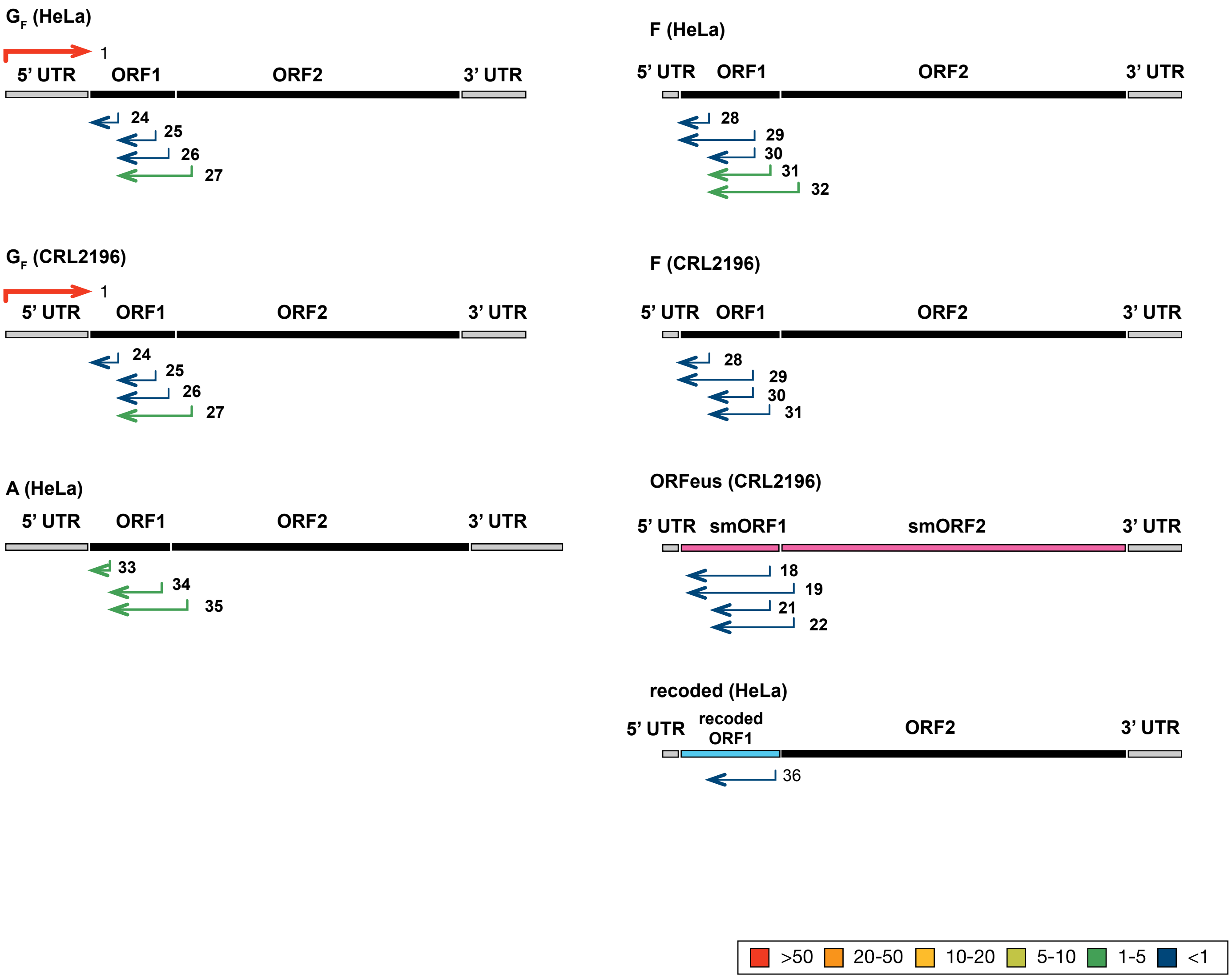

FIg. S1 Li et al.

```
                           GATA-3                                                   Oct-1      S8
L1 TF    2823 - TTCGTGGAGAGATAATGCGTGAATTTGGTTTTGTCGTGGAATACTTTGGTTTCTCCCTCTATGATAATTGAGAGTTTGGCTGGGTATAGTAGCCTGGGCTGCAGTTTGTGTTCTCTTAGT
L1 GF                TTCGTGGAGAGATAATGGGTGAATTTAGTTTTGTCGTGGAATACTTTGGTTTCTCCATCTATGGTAATTGAGAGTTTGGCTGGGTATAGTAGCCTGGGCTGGCATTTATGTTCTCTTAGT
L1 A                 TTCGTGGAAAGATATTGTGTGAACTTGGTTTTGTCGTGGAATACTTTGGTTTCTCCATCTATGGTAATTGAGAGTTTGGCCGGGTATAGTAGCCTGGGCTGGCATTTGTGTTCTCTTAGT
L1 FII               TTCGTGGAAAGATAATGTGTGAATTTGGTTTTGTCGTGGAATACTTTGGTTTCTCCATCTATGGTAATTGAGAGTTTGGCTGGGTATAGTAGCCTGGGCTGGCATTTGTGTTCTCTTAGT
smL1                TTGGTGCTCAGGTAGTGGGTGAACTTGGTCTTGTCGTGGAACACCTTGGTCTCGCCCTCGATGATGATGCTCAGCTTGGCGGGGTACAGCAGGCGGGGCTGCAGCTTGTGCTCGCGCAGG
recoded (pJL3)      TTTGTTGAAAGGTAGTGTGTAAACTTCGTCTTATCATGAAAAACCTTAGTCTCACCTTCAATAATTATACTCAGCTTTGCAGGATAAAGGAGTCGAGGTTGTAACTTATGCTCACGTAAA
               ** **    ** ** ** ** ** ** ** ** ** ** ** ** ** ** ** ** ** ** **  * **    ** ** ** ** ** ** ** ** *  ** **    ** ** ** *  *

                                                                                            GATA-2
                                                                                        cdxA              cdxA
L1 TF    2703 - GTCTGTATAACATCTGTCCAGGCTCTTCTGGCTTTCATAGTCTCTGGTGAAAAATCTGGTGTAATTCTGATAGGCTTGCCTTTATATGTTACTTGACCTTTTTCCCTTACTGCTTTTAGT
L1 GF                GTCTGTATAACATCTGACCAGGCTCTTCTGGCTTTCATAGTCTCTGGTGAAAAATCTGGTGTAATTCTGATAGGCTTGCCTTTATATGTTACTTGACCTTTTTCCCTTACTGCTTTTAGT
L1 A                 GTCTGTATAACATCTGTCCAGGCTCTTCTGGCTTTCATAGTCTCTGGTGAAAAGTCTGGTGTAATTCTGATAGGCCTTCCTTTATATGTTACTTGACCTTTCTCCCTTACTGCTTTTAAT
L1 FII               GTCTGTATAACATCTGTCCAGGCTCTTCTGGCTTTCATAGTCTCTGGTGAAAAATCTGGTGTAATTCTGATAGGCTTGCCTTTATATGTTACTTGACCTTTTTCCCTTACTGCTTTTAAT
smL1                GTCTGGATCACGTCGGTCCAGGCGCGGCGGGCCTTCATGGTCTCGGGGCTGAAGTCGGGGTGATGCGGATGGGCTTGCCCTTGTAGGTCACCTGGCCCTTCTCGCGCACGGCCTTCAGG
recoded (pJL3)      GTTTGAATTACGTCAGTCCATGCGCGACGTGCCTTCATTGTTTCAGGACTGAAGTCAGGAGTTATGCGTATTGGTTTACCCTTGTAAGTAACCTGTCCCTTCTCGCGAACAGCCTTAAGG
               ** ** ** ** ** * ** ** *  *  ** ***** ** ** **    ** ** ** ** ** *  ** **  * ** ** ** ** ** ** ** ** ** *  ** ** ** *

                cdxA                                  Oct-1                    SRY
L1 TF    2583 - ATTCTATCTTTATTTAGTGCATTTGATGTTCTGATTATTATGTGTCGGGAGGAATTTCTTTTCTGGTCCAGTCTATTTGGAGTTCTGTAGGCTTCTTGTATGTTCATATGCATCTCATTC
L1 GF                ATTCTATCTTTATTTAGTGCATTTGGTGTTCTGATTATTATGTGTCGGGAGGAATTTCTTTTCTGGTCCAGTCTATTTGGAGTTCTGTAGGCTTCTTGTATGTTCATGGGCATCTCTTTC
L1 A                 ATTCTATCTTTATTTAGTGCATTTGTTGTTCTGATTATTATGTGTCGGGAGGAATTTCTTTTCTGGTCCAGTCTATTTGGAGTTCTGTATGCTTCTTGTATGATCATGGGCATCTCTTTT
L1 FII               ATTCTATCTTTATTTAGTGCATTTGTTGTTCTGATTATTATGTGTCGGGAGGAATTTCTTTTCTGGTCCAGTCTATTTGGAGTTCTGTAGGCTTCTTGTATGTTCATGGGCATCTCTTTC
smL1                ATGCGGTCCTTGTTCAGGGCGTTGCTGGTGCGGATGATGATGTGTCTAGAGCTGTTGCGCTTCTGGTCCAGGCGGTTGGGGTGCGGTAGGCCTCCTGGATGTTCATGTGCATCTCGTTC
recoded (pJL3)      ATTCGGTCCTTGTTAAGAGCGTTGCTAGTACGAATGATAATATGACGACTAGAGTTACGCTTTTGATCAAGCCTGTTGGGTGTACGATATGCCTCCTGAATATTCATGTGCATTTCGTTT
               ** *  ** ** ** ** ** **    ** *  ** ** ** ** *        ** *  ** ** ** ** *  ** ** ** *  ** ** ** ** **  ****  **** ** **

                    c/EBP
L1 TF    2463 - TTTAGATTTGGGAAGTTTTCTTCAATAATTTTGTTGAAGATGTTTGCTGGACCTTTGAGTTGAAAATCTTCATTCTCATCCACTCCTATTATCCGTACGTTTGGTCTTCTTATTGTGTCC
L1 GF                TTTAGATTTGGGAAGTTTTCTTCAATAATTTTGTTGAAGATGTTTGCTGGTCCTTTGAGTTGAAAATCTTCATTCTCATCCACTCCTATTATCCGTAGGTTTGGTCTTCTCATTGTGTCC
L1 A                 TTTATGTTTGGGAAGTTTTCTTCTATTATTTTGTTGAAGATATTAGCTGGCCCTTTAAGTTGAAAATCTTCATTCTCATCAATTCCTATTATCCGTAGGTTTGGTCTTCTCATTGTGTCC
L1 FII               TTTAGGTTTGGGAAGTTTTCTTCTATAATTTTGTTGAAGATATTTGCTGGTCCTTTGAGTTGAAAATCTTCATTCTCATCTACTCCTATTATCCGTAGGTTTGGTCTTCTCATTGTGTCC
smL1                TTCAGGTTGGGGAAGTTCTCCTCGATGATCTTGTTGAAGATGTTGGCGGGGCCCTTCAGCTGGAAGTCCTCGTTCTCGTCCACGCCGATGATGCGCACGTTGGGGCGGCGGATGGTGTCC
recoded (pJL3)      TTAAGGTTAGGAAAATTCTCCTCTATTATCTTATTAAAAATATTAGCCGGTCCCTTTAACTGGAAGTCCTCGTTTTCGTCAACACCAATAATTCTAACATTAGGTCGCCTGATAGTATCT
               ** *  ** ** ** ** ** ** ** ** ** ** ** ** ** ** ** ** ** ** ** *  ** ** ** ** ** ** ** ** *  ** ** ** *  *  ** ** *  *  ** ** **

                                                                  SRY  Pbx-1                   HSF2/HSF2   ΔE
L1 TF    2343 - TGGATTTCCTGGATATTTTGAGTTAGGATCTTTTTGCATTTTCCATTTTCTTTGATTGTTGTGCCGATGTTCTCTATGGAATCTTCTGCACCTGAGATTCTCTCTTCCATCTCTTGTATT
L1 GF                TGGATTTCCTGGATGTTTTGAGTTAGGATCTTTTTGCATTTTCCATTTTCTTTGATTGTTGTGCCGATGTTCTCTATGGAGTCTTCTGCACCTGAGATTCTCTCTTCCATCTCTTGTATT
L1 A                 TGGATTACCTGGATGTTTTGAGTTAGGATCCTTTTGCATTTTGTATTTTCTTTGACTGTTGTGTCGATGTTCTCTATGGAATCTTCTGCACCTGAGATTCTCTCTTCCATTTCTTGTATT
L1 FII               TGGATTTCCTGGATGTTTTGAGTTAGGATCTTTTTGCATTTTCCATTTTCTTTGATTGTTGTGCCGATGTTCTCTATGGAATCTTCTGCACCTGAGATTCTCTCTTCCATCTCTTGTATT
smL1                TGGATCTCCTGGATGTTCTGGGTCAGGATCTTCTTGCACTTGCCGTTCTCCTTGATGGTGGTGCCGATGTTCTCGATGCTGTCCTCGGCGCCGCTGATGCGCTCCTCCATCTCCTGGATG
recoded (pJL3)      TGAATCTCTTGTATGTTCTGGGTAAGTATTTTCTTACACTTACCGTTCTCCTTTATAGTAGTTCCTATATTTCAATAGAGTGCTCAGCTCCAGATATTCGTTCCTCCATTTCCTGGATG
               ** **  * ** ** ** ** ** ** ** **  * ** ** **    ** ** ** *  ** **  * ** ** ** **     *  ** ** **    ** *  ** ***** ** ** **

                                                    SRY                                               SRY
L1 TF    2223 - CTGTTGCTGATGCTCAAATCTATGGTTCCAGATTTCTTTCCTAGGGTTTCTATCTCTAGTGTTGCCTCGCTTTGAGTTTTCTTTATTGTGTCTACTTCC - 2125 (AS)
L1 GF                CTGTTGCTGATGCTGGCATCTATGGTTCCAGATTTCTTTCCTAGGGTTTCTATCTCCAGCGTCGCCTCACTTTGGGTTTTCTTTATTGTGTCTACTTCC
L1 A                 CTGTTGCTGATGCTCGCATCTATGGTTCCAGATCTCTTTCCTAGGGTTTCTATCTCCAGCGTTGCCTCGCTTTGGGTTTTCTTTATTGTGTCTACTTCC
L1 FII               CTGTTGCTGATGCTCGCATCTATGGTTCCAGATTTCTTTCCTAGGGTTTCTATCTCCAGCGTTGCCTCACTTTGGGTTTTCTTTATTGTGTCTACTTCC
smL1                CGGTTGCTGATGCTCAGGTCGATGGTGCCGCTCTTCTTGCCCAGGGTCTCGATCTCCAGGGTGGCCTCGCTCTGGGTCTTCTTGATGGTGTCGACCTCG
recoded (pJL3)      CGATTCGAAATGGAGAGGTCAATAGTACCGCTCTTTTTACCAAGAGTCTCAATTTCAAGAGTAGCTTCCGACTGCGTCTTTTTAATCGTATCAACCTCG
               *  **    ***      ** ** ** **     * ** ** ** ** ** ** ** ** ** ** ** **    ** ** ** ** ** ** ** ** **
```

Fig. S2A - Li et al

B

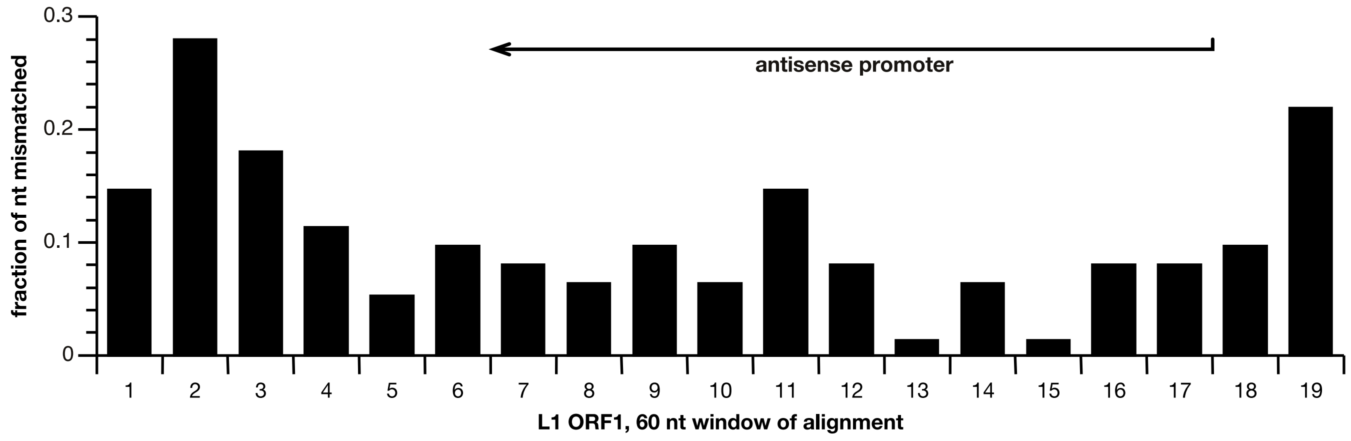

FIg. S2 B, Li et al.

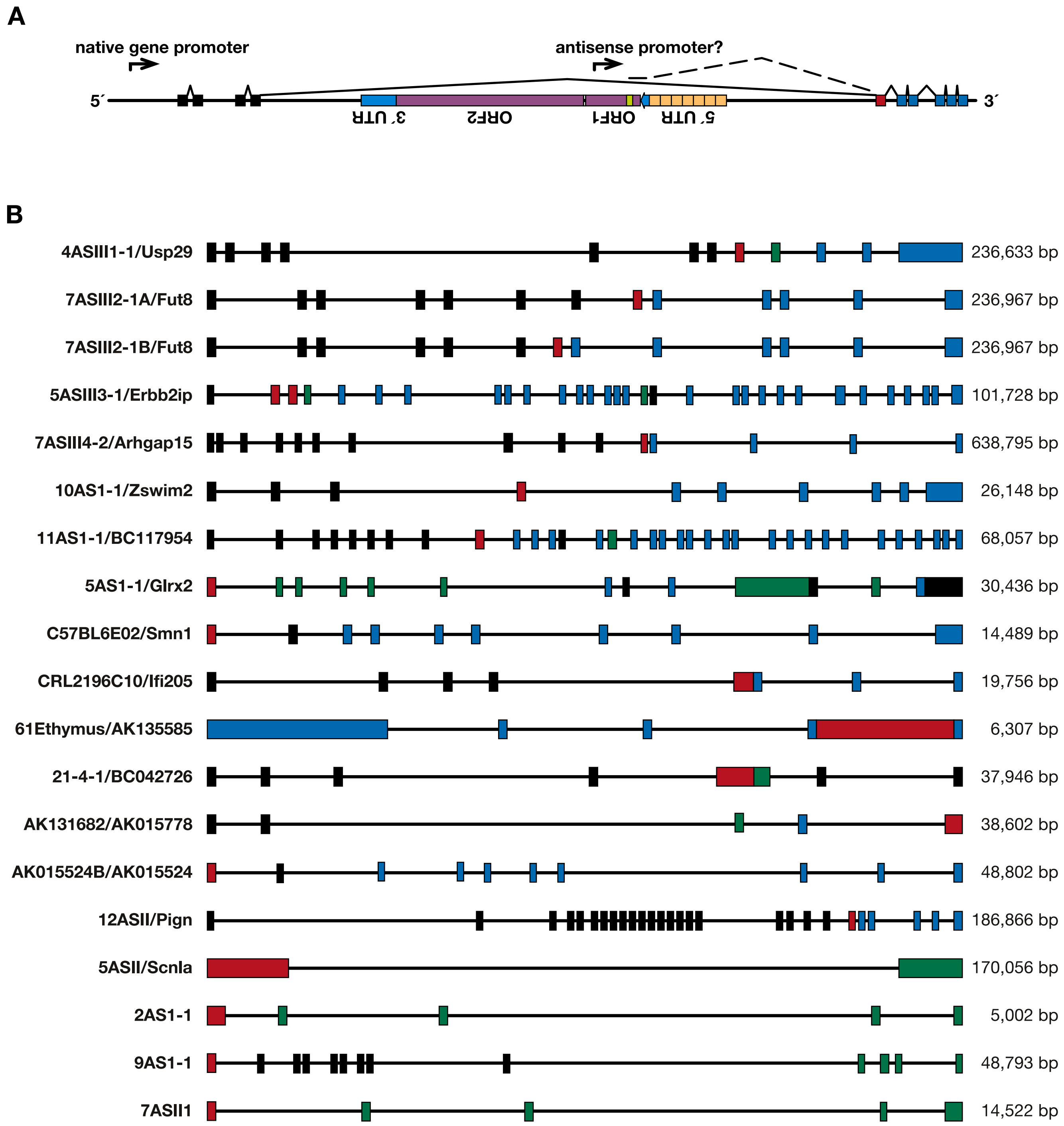

Fig. S3 Li et al.

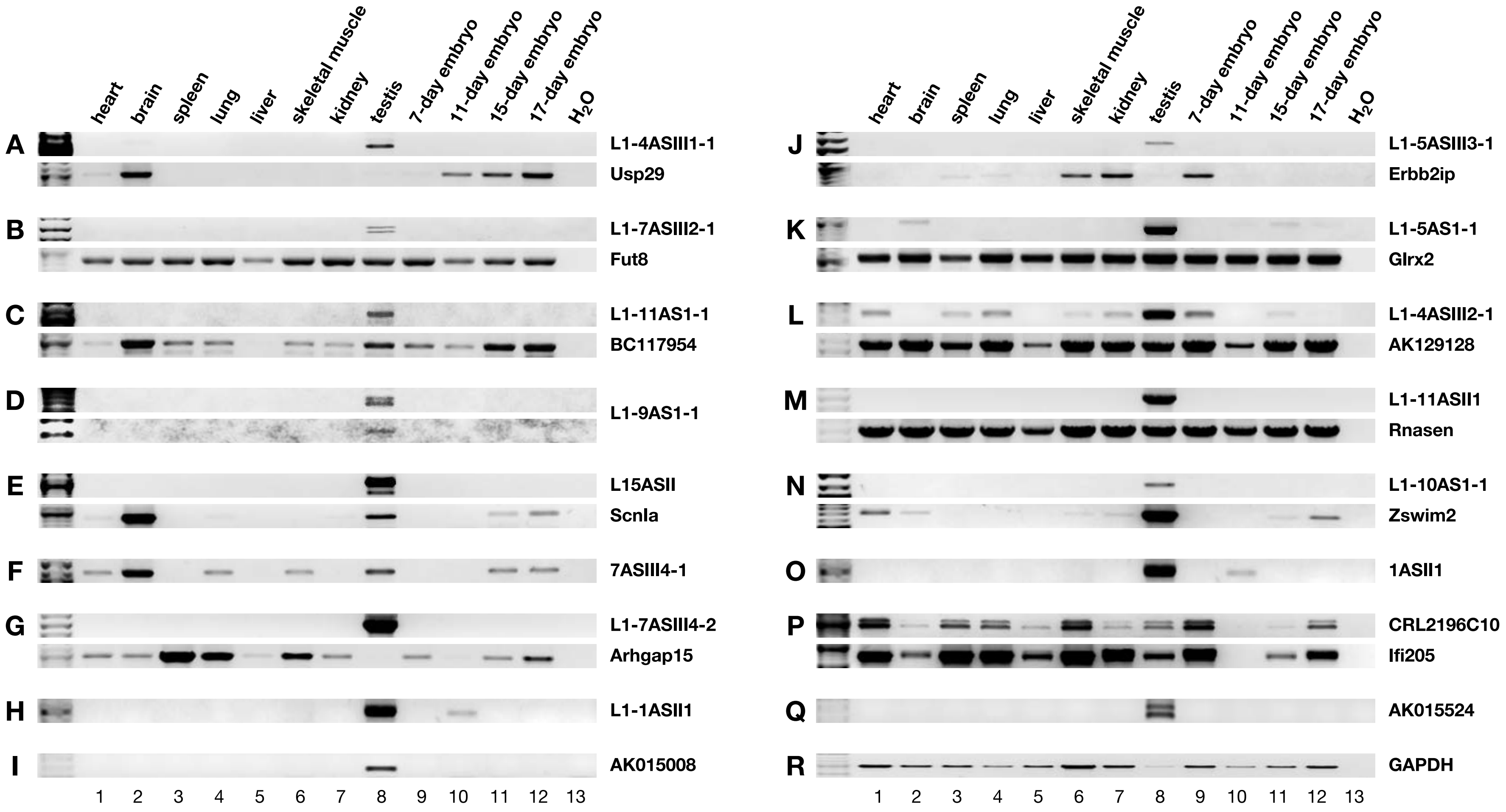

Fig. S4 Li et al.

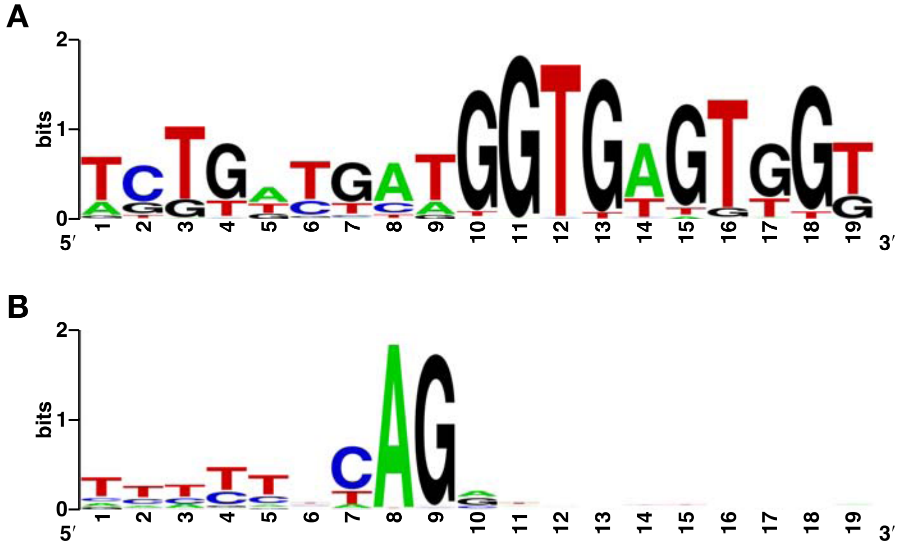

Fig. S5 - Li et al.

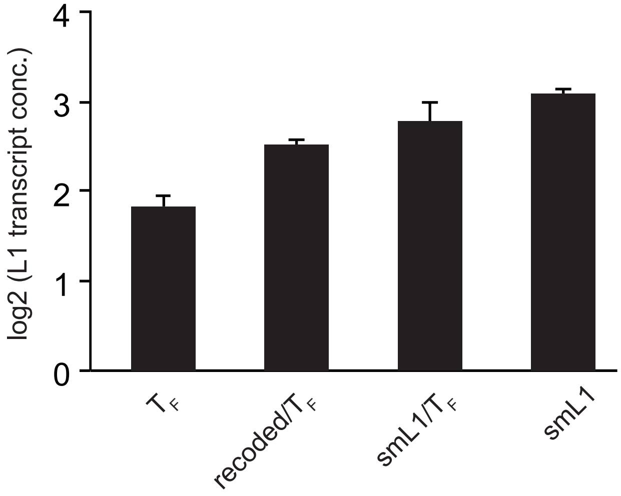

FIg. S6 Li et al.

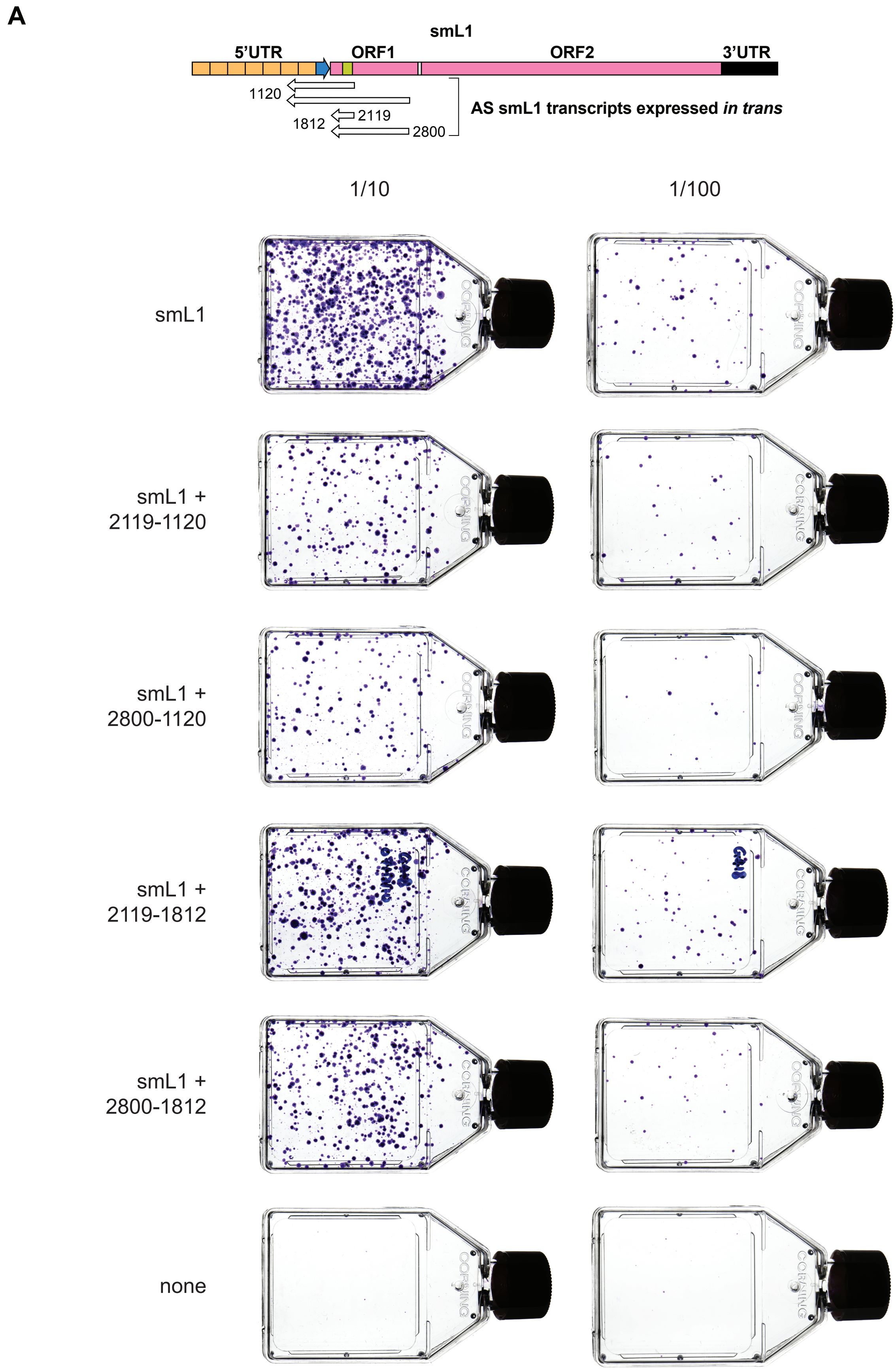
A
smL1
5'UTR
ORF1
ORF2
3'UTR
1120
2119
1812
2800
AS smL1 transcripts expressed *in trans*
1/10
1/100
smL1
smL1 +
2119-1120
smL1 +
2800-1120
smL1 +
2119-1812
smL1 +
2800-1812
none

Supp. Fig. 7A

B

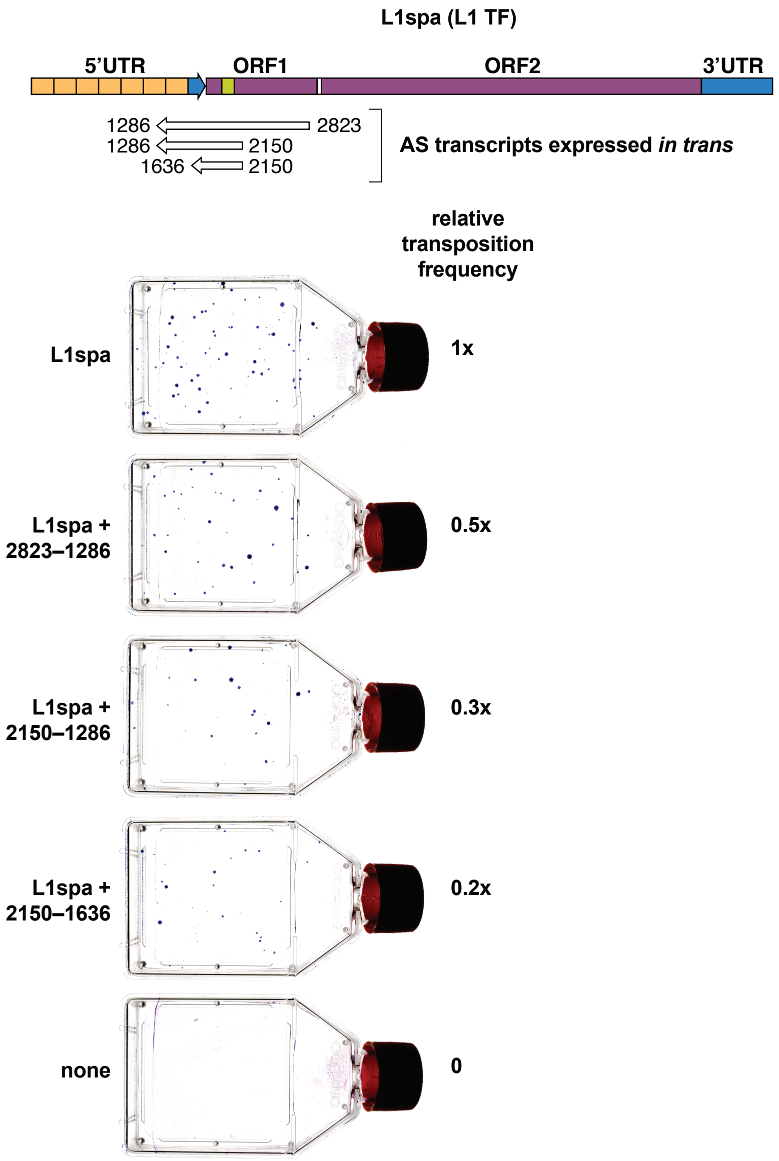
L1spa (L1 TF)
5'UTR
ORF1
ORF2
3'UTR
1286
2823
1286
2150
1636
2150
AS transcripts expressed *in trans*
relative transposition frequency
L1spa
1x
L1spa + 2823–1286
0.5x
L1spa + 2150–1286
0.3x
L1spa + 2150–1636
0.2x
none
0

Supp. Fig. 7B

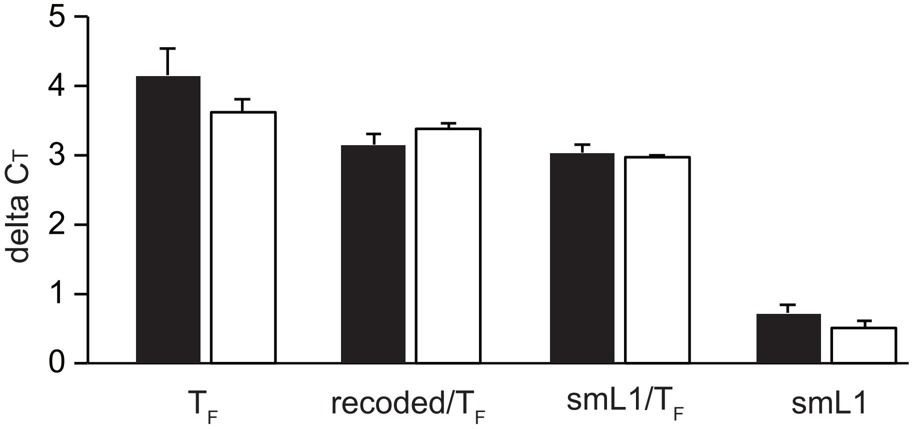

Fig. S8 Li et al.
